# A Bayesian Inference Framework to Reconstruct Transmission Trees Using Epidemiological and Genetic Data

**DOI:** 10.1371/journal.pcbi.1002768

**Published:** 2012-11-15

**Authors:** Marco J. Morelli, Gaël Thébaud, Joël Chadœuf, Donald P. King, Daniel T. Haydon, Samuel Soubeyrand

**Affiliations:** 1 Institute of Biodiversity, Animal Health and Comparative Medicine, College of Medical, Veterinary and Life Sciences, University of Glasgow, Glasgow, United Kingdom; 2 INRA, UMR BGPI, Cirad TA A-54/K, Montpellier, France; 3 INRA, UR546 Biostatistics and Spatial Processes, Avignon, France; 4 Institute for Animal Health, Pirbright, United Kingdom; Imperial College London, United Kingdom

## Abstract

The accurate identification of the route of transmission taken by an infectious agent through a host population is critical to understanding its epidemiology and informing measures for its control. However, reconstruction of transmission routes during an epidemic is often an underdetermined problem: data about the location and timings of infections can be incomplete, inaccurate, and compatible with a large number of different transmission scenarios. For fast-evolving pathogens like RNA viruses, inference can be strengthened by using genetic data, nowadays easily and affordably generated. However, significant statistical challenges remain to be overcome in the full integration of these different data types if transmission trees are to be reliably estimated. We present here a framework leading to a bayesian inference scheme that combines genetic and epidemiological data, able to reconstruct most likely transmission patterns and infection dates. After testing our approach with simulated data, we apply the method to two UK epidemics of Foot-and-Mouth Disease Virus (FMDV): the 2007 outbreak, and a subset of the large 2001 epidemic. In the first case, we are able to confirm the role of a specific premise as the link between the two phases of the epidemics, while transmissions more densely clustered in space and time remain harder to resolve. When we consider data collected from the 2001 epidemic during a time of national emergency, our inference scheme robustly infers transmission chains, and uncovers the presence of undetected premises, thus providing a useful tool for epidemiological studies in real time. The generation of genetic data is becoming routine in epidemiological investigations, but the development of analytical tools maximizing the value of these data remains a priority. Our method, while applied here in the context of FMDV, is general and with slight modification can be used in any situation where both spatiotemporal and genetic data are available.

## Author Summary

In order to most effectively control the spread of an infectious disease, we need to better understand how pathogens spread within a host population, yet this is something we know remarkably little about. Cases close together in their locations and timing are often thought to be linked, but timings and locations alone are usually consistent with many different scenarios of who-infected-who. The genome of many pathogens evolves so quickly relative to the rate that they are transmitted, that even over single short epidemics we can identify which hosts contain pathogens that are most closely related to each other. This information is valuable because when combined with the spatial and timing data it should help us infer more reliably who-transmitted-to-who over the course of a disease outbreak. However, doing this so that these three different lines of evidence are appropriately weighted and interpreted remains a major statistical challenge. In our paper we present a new statistical method for combining these different types of data and estimating trees that show how infection was most likely transmitted between individuals in a host population. Because sequencing genetic material has become so affordable, we think methods like ours will become very important for future epidemiology.

## Introduction

Predicting the most likely transmission routes of a pathogen through a population during an epidemic outbreak provides valuable information, which can be used to inform intervention strategies and design control policies [1], [2]. In principle, studying transmission routes during past epidemics is likely to be broadly informative of how the same pathogens spread through similar populations in future outbreaks. Estimating a set of connected transmission routes from a single case is synonymous with estimating the transmission tree corresponding to the outbreak. Uncovering the transmission routes between individual hosts or other relevant infectious units (for example farms or premises) can provide valuable epidemiological information, such as the factors associated with source and target individuals, dissemination kernels and transmission modes. Unfortunately, reconstructing these transmission trees with available data can be an exceptionally hard task, as the problem is typically underdetermined: the precise number of cases is often unknown, and dates and times of infections are rarely known with precision, making it difficult to distinguish between a large number of alternative scenarios [3].

With knowledge of location and timing of disease incidence it is possible to sample transmission trees that are consistent with the space-time data, and when these samples of trees share emergent statistical or structural properties, they can lead to epidemiological insights. For example, Haydon *et al.* [4] generated transmission trees corresponding to the 2001 Foot-and-Mouth Disease Virus (FMDV) epidemics in the UK, and used these trees to estimate the reproductive number during different weeks of the epidemic. These trees could be pruned to investigate the consequences of different or earlier interventions on the final size of the epidemics. However, the data were consistent with very large numbers of different trees and so the approach was not suited to identifying with confidence “who infected who”.

For pathogens with high mutation rates that fix mutations across their genome during the course of a single outbreak, genetic data can provide critical additional information regarding the relationships between isolates. The last few years have witnessed a revolution in our ability to generate genomic data relatively cheaply and in an automatised fashion [5]. Pathogen genome sequences collected during epidemics, if sufficiently diverse, can then be used to discriminate between alternative transmission routes.

Several attempts to reconstruct transmission pathways have tried to combine genetic and other epidemiological data, many by adding spatial or temporal information to the process of phylogenetic reconstruction [6]–[11]. However, Jombart *et al.* point out that a “phylogenetic” approach attempts to infer hypothetical common ancestors among the sampled genomes, and may not be appropriate for a set of genomes containing both ancestors and their descendants [12]. Cottam *et al.* [13] identified a large set of transmission trees that were consistent with available genetic data, and ranked the likelihood of these trees using data on their relative timings, to find the most likely transmission tree. Ypma *et al.* [14] moved this approach forward by constructing an inference scheme that uses spatial, temporal and genetic data simultaneously, but assumed these data are independent of each other. Genetic and epidemiological data are evidently correlated, and a rigorous inference scheme should estimate the likelihood of a transmission tree accounting for these correlations.

**Figure 1:**
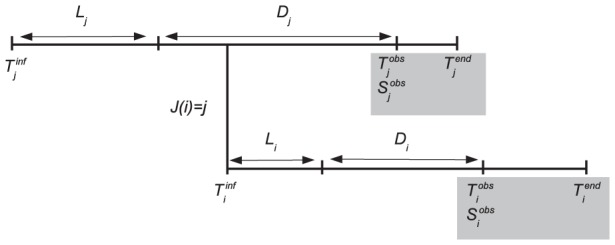
Dynamical model of pathogen transmission between a source premise 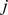 and a receptor premise 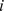. Premises are considered confined at fixed locations in space. Variables covered by the grey rectangles are observable. A premise 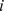 is infected at time 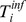, becomes infectious after a latent period 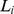, is observed at time 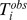, when a viral sequence 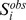 is obtained, and is removed from susceptible population (*i.e.* loses its ability to infect other premises) at time 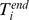. When an infected premise is reported, the duration period from infectiousness to detection, 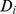, is assessed by experts based on symptom observation. This assessment is called 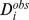.

In this work, we present a novel framework, based on a bayesian inference scheme, able to reconstruct transmission trees and infection dates of susceptible premises, integrating coherently genetic and spatiotemporal data with a single model and likelihood function. Our scheme uses epidemiological data (times of reporting and removal from the susceptible population of infected, spatially-confined hosts, their locations, and estimates of the age of an infection based on clinical signs) together with pathogen sequences obtained from infected hosts to estimate transmission trees and infection dates during outbreaks. The genetic information is incorporated considering the probability distribution of the number of substitutions between sequences during the time durations separating them, and computing the likelihood of observing these sequences for a given transmission tree and the estimated infection dates. Each host generates an isotropic infectious potential responsible for transmission between hosts, whose strength is estimated from the data; the dynamical progression of the disease, from latency to infectiousness is part of the estimation scheme (for a visual representation see Fig. 1).

As an illustration of the method, we concentrate on the case of FMDV, an infectious disease affecting cloven-hoofed animals, which has severely affected the UK in 2001 and, on a smaller scale but still contentiously, in 2007. The infectious agent is single-stranded, positive-sense RNA virus, belonging to the genus *Aphthovirus* in the *Picornaviridae* family, and its small genome (8.2 kb) is easily sequenced. Its high substitution rate (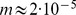 per nt per day as measured over part of the 2001 UK epidemic [13]), implies that the number of mutations accumulate during infection of host individuals on a single premise is sufficient to be reasonably confident of distinguishing between infected premises. Upon infection by FMDV, a host individual first experiences a non-infectious latent period with lesions appearing on peripheral epithelia subsequently. The virus can spread through aerosol dispersal, on fomites, or through direct contact. Importantly, a visual exam of the clinical state of the lesions on infected hosts can provide valuable information about the age of the infection. For this application, premises comprising populations of spatially-confined hosts will be considered as the unit of infection (the centroids of premises will be used as geographical coordinates), and complete FMDV genomes sampled from each premise will be used for the inference; the removal of a premise from the population corresponds to its culling. As the time course of FMDV infection within an individual host follows empirically characterised distributions [13], when transmission events are inferred between premises infected at very different times and therefore with correspondingly long and unrealistic apparent latency durations, we interpret these as an indication of the presence of one or more unsampled infected premises, that epidemiologically linked the observed premises.

After testing our method on simulated data, we considered two real datasets from two different FMDV epidemics: the 2007 UK epidemic (8 premises) [15] and the Darlington cluster within the 2001 UK epidemic (15 premises) [13]. For the former case, we confirmed the role of IP5 as the link between the two phases of the epidemics, whereas for the latter, our scheme highlights the presence of premises outside our sample that were part of the transmission process. While in this paper we discuss results related to FMDV, our method is in principle general and can be applied to epidemics generated by other pathogens, for which genetic and epidemiological data are both available.

## Results

### Assessment of the method with a test outbreak

Prior to applying our method to real data, we first used our model to simulate data for an outbreak infecting 20 premises whose locations are known in a 22×11 km area. The model was fitted to the observable data, that is, for each premise 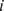, the time 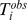 at which the virus was detected, a 8000 bp DNA sequence 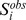 sampled at 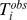, an assessment of the lesion age 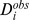, and the time 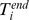 at which the premise was culled (see Fig. 1 for a visualisation). More information on this dataset can be found in Text S1.

**Figure 2:**
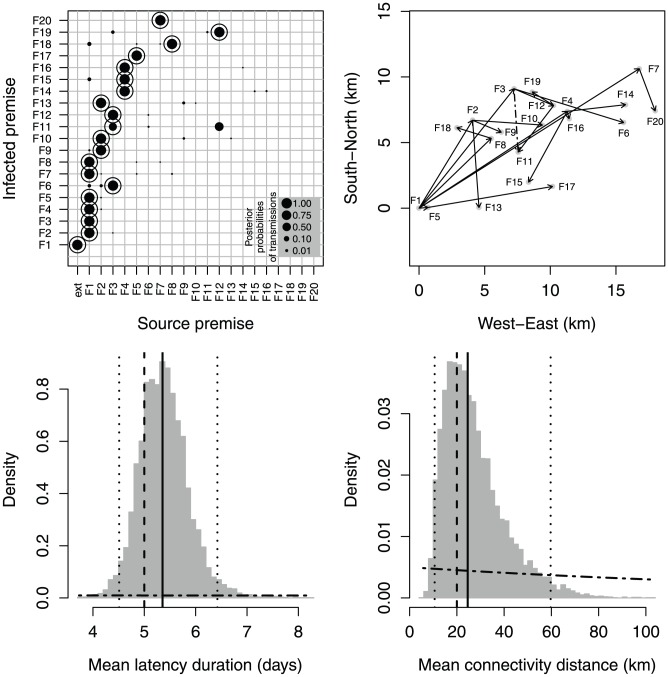
Estimation output for the simulated outbreak. Top left: true transmissions (circles) and posterior probabilities of transmissions (dot sizes are proportional to probabilities). Top right: tree with the highest posterior probability (solid arrows); Only transmission 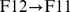 is not consistent with the true tree (the true transmission is 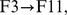 dashed arrow). Bottom: posterior distributions (histograms) of mean latency duration (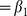; left) and mean transmission distance (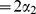; right); dashed lines: true values; dotted-dashed curves: prior distributions; solid lines: posterior medians; dotted lines: posterior quantiles 0.025 and 0.975.

In Fig. 2 (top left), the size of the dots corresponds to the posterior probabilities of pairwise transmissions, while the circles represent the true transmissions as they occurred in the simulation. Fig. 2 (top right) shows the tree with highest posterior probability. We note that only one true transmission (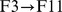) is not reconstructed accurately, the algorithm instead identifying 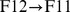. However, the 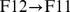 transmission has a high posterior probability and is included in the tree with the second highest posterior probability (see Fig. S2). The posterior probabilities for the mean latency duration and the mean transmission distance include the true values in the 95%-posterior intervals (bottom panels of Fig. 2). Posterior distributions for other model parameters and latent variables are provided in the Figs. S3, S4.

In order to test our method for a large dataset, we considered an upscaled simulation of an outbreak infecting 100 premises. Results are described in Text S1.

### An outbreak with two phases – 2007 FMDV in UK

**Figure 3:**
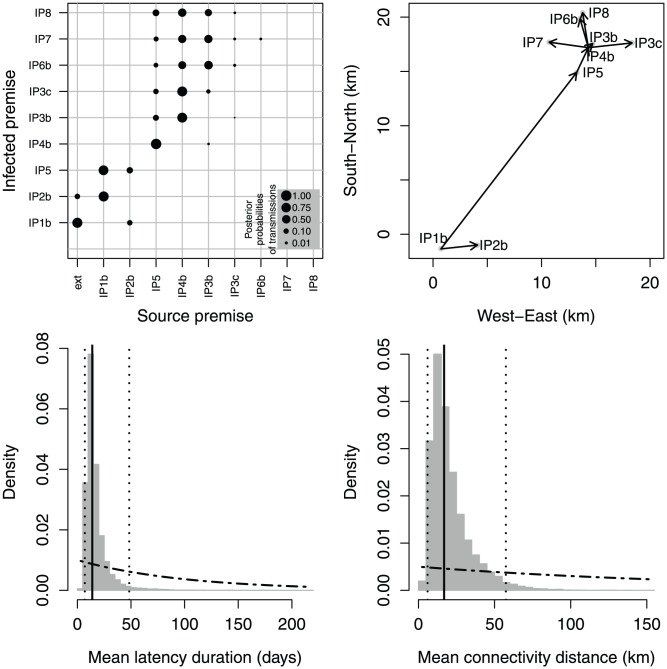
Estimation output for the 2007 UK outbreak. Top left: posterior probabilities of transmissions (dot sizes proportional to probabilities). Top right: tree with the highest posterior probability mapped in space (black arrows). Bottom: posterior distributions (histograms) of mean latency duration (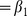; left) and mean transmission distance (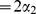; right); dotted-dashed curves: prior distributions; solid lines: posterior medians; dotted lines: posterior quantiles 0.025 and 0.975.

Having established the validity of the inference scheme, we applied it to a dataset corresponding to the 2007 outbreak of FMDV in the UK, which infected 8 premises in Surrey and Berkshire [15]. Genetic sequences and epidemiological collected on each premise are available in the Dataset S1 and S2, respectively. The most likely reconstructed scenario (Fig. 3, top right) comprises two phases: IP1b was infected by an external source, and transmitted the virus to the neighbouring premise IP2b and to IP5 further away; the virus remained contained and undetected on IP5 until it spread to a closeby premise IP4b; finally the virus spread from IP4b to the other premises. While the link made by IP5 between the two phases is highly supported, the estimation of the other transmissions was more uncertain: within the two clusters (IP1b, IP2b, IP5) and (IP5, IP4b, IP3b, IP3c, IP6b, IP7, IP8) several other transmission scenarios have non-negligible posterior probabilities (Fig. 3, top left and Fig. S5). The mean estimated latency duration has a posterior median of 14 days and a 95%-credible interval of (6, 49) (as shown in Fig. 3, bottom left); the long delay between the infection of IP5 and the subsequent transmissions is responsible for this result (posterior distributions of latency durations of every premises are shown in Fig. S7). The long distance between IP5 and its source (IP5 is 18.2 km away from IP1b) explains the large mean transmission distance (Fig. 3, bottom right), whose posterior median is 17 km and 95%-posterior interval is (5,58). Posterior distributions of other model parameters and latent variables are provided in Figs. S6, S7, while a phylogenetic tree, based on statistical parsimony tree, implemented in the software package TCS [16] is represented in Fig. S14.

### A cluster with independent introductions – 2001 FMDV in UK (Durham county)

For a more complex scenario, we considered the FMDV epidemic that occurred in the UK in 2001, and in particular a group of 12 premises within the so-called “Darlington cluster” (Durham county), for which one virus sequence per premise is available [13]. This spatial cluster comprises 3 additional premises that were not epidemiologically linked to the rest of the cluster and which we exclude (we discuss the choice of the subgroup of premises in the Text S1). Genetic sequences and epidemiological data for this cluster can be found in the Datasets S3 and S4, respectively.

**Figure 4:**
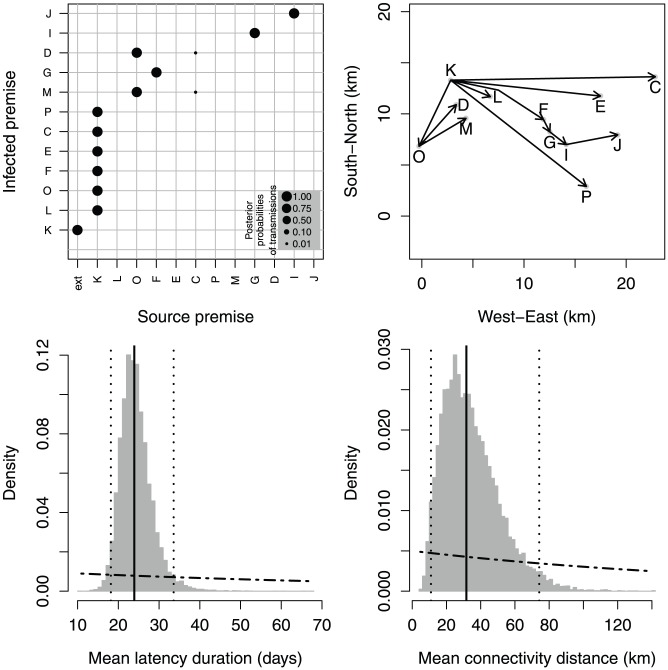
Estimation output for the 2001 UK outbreak (Darlington cluster). Top left: posterior probabilities of transmissions (dot sizes proportional to probabilities). Top right: tree with the highest posterior probability mapped in space (black arrows). Bottom: posterior distributions (histograms) of mean latency duration (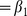; left) and mean transmission distance (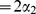; right); dotted-dashed curves: prior distributions; solid lines: posterior medians; dotted lines: posterior quantiles 0.025 and 0.975.

Our method allowed us to reconstruct a transmission scenario with little ambiguity, accounting for over 99% of the posterior probability, where premise K plays the role of a hub and only two chains of transmissions of length greater than two are found (Fig. 4, top panels). When premises become infectious approximately at the same time, they have a very low probability of mutual infection, even if the collected genomes are very close and share substitutions (premises M and D, or L and E, for example). Premise K, on the other hand, became infectious very early on and is then estimated to have seeded the infection to the many premises that were observed at later times.

Interestingly, some premises infected by the hub share mutations that are not found on the other premises, suggesting that different unsampled strains evolved on the hub and went on to infect distinct clusters of farms (see the statistical parsimony network in Fig. S14). However, another hypothesis can be formulated: the virus fixed the common substitutions while replicating on an unsampled premise, which constitutes a missing node in the transmission tree. This “ghost premise” went on to infect the premises we observed. The missing node scenario is supported by the distribution of the mean latency duration estimated for this dataset, which has a median of 24 days, and a 95%-posterior interval of (17, 35) (Fig. 4, bottom left). These values are inconsistent with a typical latency period of FMDV of 5 days (95% confidence interval of 1–12) [17]–[19]. In particular, the premises infected by the hub all display high mean latency values (Fig. S11). We propose that these unrealistically long latency periods indicate the existence of missing premises intermediate in the chain of infection and so in our model, latency should be considered as an aggregated parameter, corresponding to the the sum of the real latent period and the time the virus spent on the unsampled premise. We will return to this point in the Discussion. The comparison of our results with those found by Cottam *et al.* on the same dataset [13] highlights that our method strengthens the role of infecting hubs in the network (premise K), and therefore infers a lower number of long transmission chains. Details about the individual differences between the most likely trees inferred by the two methods can be found in Text S1, while transmission trees with higher posterior probabilities and posterior probabilities of other paramteres can be found in Figs. S9, S10.

### Spatial connectivity

The estimates of the transmission kernel for the two real data sets are similar: the 95%-posterior intervals of the mean transmission distance (defined as 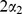) overlap, ranging from 5 to 58 km for the 2007 outbreak and ranging from 9 to 72 km for the 2001 epidemic (Figs. 3 and 4, bottom right panels). On the other hand, the posterior distributions we obtained are related to the range of distances covered in the data sets (up to about 24 km for 2007 and 16 km for 2001), and cannot be used to extrapolate long distance transmission events: despite the large values of the mean transmission distance, the lengths of the average inferred transmission in the trees with the highest posterior probabilities are 4.3 km for the 2007 outbreak and 5.8 km for the 2001 epidemic.

### Sensitivity of the inference to the uncertainty of lesion aging

In the inference scheme, we used vague priors for model parameters. When we estimated the interval from the end of latency to detection, however, we used a more informative prior, centered over the estimated lesion age (Eq. (8) in Materials and Methods). We investigated the effect on the most likely transmission tree of (i) using a flatter prior (thus believing less than we did previously in the veterinarian assessment) and (ii) using a more peaked prior (thus believing in it more). The trees are illustrated in Fig. S12, and the priors in the Fig. S13. For the 2007 outbreak, the tree differed only by one transmission in case (i), and by three transmissions in case (ii). Remarkably, in all cases, the identification of the link between the two phases in IP5 maintained a posterior probability of one. For the 2001 epidemic, the star-like shape (with K as a hub) of the tree was strengthened in case (i), where premise K now infected 9 premises, while more chains of length greater than two were inferred in case (ii). Constraining the inference less around the estimates of the lesion ages relaxes the timing constraints and increases the weight accorded to genetic similarity in the transmission inference. As a result, transmissions mirror more closely the phylogenetic structure of the dataset, leading to a reduced hub role of premise K. In conclusion, we remark that the tree structure is robust and does not crucially depend on the specific choice of the prior for the values of the time intervals between the end of latency and detection (lesion ages).

### Performance assessed over series of simulations

Our method relies on one approximation: we do not reconstruct the genomes transmitted at the times of infection, and therefore we obtain a pseudo-posterior probability for the genetic data, where the similarity between isolates only depends on the Hamming distance between the sequences, and not on the full genetic network (see Materials and Methods for details). We checked whether the use of a pseudo-posterior distribution led to appropriate inference by applying the estimation algorithm to three series of 100 simulations (one for the test outbreak and two for the FMDV datasets) generated using our model. For the first series, we used the parameter values that were used in the test simulation. For the two other series, we used the posterior medians of the parameters estimated previously. We were especially interested in the fraction of correctly predicted pairwise transmissions: for each premise, between 79% and 93% of the simulations reproduced the source with the highest posterior probability in the original inference (Table 1). Given the challenging nature of the data sets (closely spaced premises becoming infectious almost simultaneously in the test data, and an abnormally long period of time between infection and transmission between two waves of infection in the 2007 data), these results suggest the approximation is performing well. Moreover, the mean of the posterior probability of each true transmission (the proportion of iterations in the chain at which a premise is infected by the estimated source) is also reproduced in about 80% of the cases. Performances vary slightly across datasets depending on the characteristics of the epidemics (e.g. number of premises and parameter values), but are broadly compatible. For example, in the second phase of the 2007 outbreak, several scenarios have high posterior probabilities, lowering the fraction of correctly estimated transmissions. Further performance estimators are listed in Table S1.

**Table 1:** Performance of the estimation algorithm over three series of 100 simulations (test, 2007, 2001).

Criterion	Test	2007	2001
Fraction (Sd.) of correct prediction of PT	0.89 (0.08)	0.79 (0.13)	0.93 (0.06)
Mean (Sd.) of post. prob. of true PT	0.85 (0.08)	0.76 (0.10)	0.93 (0.05)

The criteria used are the fraction (and standard deviation; Sd.) of correct predictions of pairwise transmissions (PT) and the mean (and Sd.) of the posterior probabilities of the true pairwise transmission.

## Discussion

We propose here a new bayesian inference scheme, with which we estimate transmission trees and infection dates for an epidemic outbreak using genetic and epidemiological data. Our scheme is general, and with slight modification can be applied to rapidly evolving pathogens affecting spatially-confined hosts. To illustrate how this approach can be used to generate new insights and deliver statistically formal measures of confidence (in particular transmission links), we applied it to the case of an RNA virus (FMDV) infecting premises whose spatial location is known. The knowledge of complete viral sequences, timing of reporting and culling of premises and estimates of the age of an infection made this case an ideal benchmark. After testing our method on simulated data (20 premises), we applied it to two pre-existing datasets: the still disputed 2007 FMDV outbreak in the UK (8 premises) [15] and the Darlington cluster within the larger 2001 epidemic (12 premises) [13]. The method proved successful in reconstructing the transmission network on the test dataset, and highlighted the role of IP5 as a relay between the two phases of the 2007 outbreak. The results for the Darlington cluster are intriguing, as they highlight the likely incompleteness of the dataset, and suggest the presence of unobserved premises in the transmission tree. The performance of the algorithm was evaluated through simulations, which showed the inference scheme to be consistent and accurate and able to deal successfully with clusters of infections.

The power of this inference platform relies on a number of simplifying assumptions. In this application we have made two in particular that require further consideration. The first postulates that the epidemics are generated by a single introduction of the pathogen to a single premise. While this may often be adequate for small or early stage outbreaks, it is likely to be inadequate for more complex cases. For example, the Darlington dataset is a small subset of the 2001 epidemic, in which it was first considered to be an isolated cluster of infected premises. Previous analysis on the whole cluster [13] demonstrated two independent introductions. Trying to estimate “polyphyletic” transmission trees assuming only a single root would strain this formulation of the model and lead to unrealistic results. In order to solve this problem, the MCMC should be able to explore a parameter space where independent introductions range from one to the number of the premises (each of them being independently infected by an external source) and compute their likelihood. Moreover, the genetic data can be used to discriminate between a situation where a single external source infects several spatially-confined hosts in a cluster, and the presence of multiple external sources, characterised by distinct genomes. In practice, we could proceed by (i) describing the external source(s) as a set of genetic sequences varying in time (and possibly in space), (ii) specifying the probability of transmission of the infection from the external source(s) to any of the premises and (iii) updating the transmission tree at each iteration of the MCMC by comparing this probability with the probability of transmission from one of the infectious premises in the cluster considered.

The second assumption is that the epidemic has been completely observed and that there are no missing nodes in the transmission tree. When this assumption is likely to be violated, as in the case of the Darlington cluster, our method inferred unrealistically long latency times for some premises, an indication that a missing intermediate infected premise, where virus might have replicated extensively, may have been involved in the transmission chain. This situation is particularly likely in large epidemics, where perfect knowledge of every case is unlikely, or in epidemics arising in areas or countries where host or premise identification is ambiguous and comprehensive collection of data not feasible. In the 2007 outbreak, where no infected premises were missing, the premise linking the two phases showed a mean latency duration of over 25 days. In this case, the observation results from the real time the virus spent on the farm prior to its detection and reporting: by the time it was observed, the animals had started to heal and dating the lesions was more difficult. The long latency times could also account for the time virus spent in a non-replicative state (e.g. on fomites): this case would be indicated by a slow rate of evolution on the premise where the virus is observed. In conclusion, extended latency times are valuable “alarm bells”, as they suggest a discrepancy between the observations and the actual course of the disease. A substantial improvement to the scheme would be to include in the inference additional sources of data, such as the locations of premises that may have maintained infections that were not detected, or premises that were infected but were removed prior to being confirmed as infected. We leave this development for future work. We only mention here that the solution given in the paragraph above to deal with multiple introductions could be adapted to deal with missing premises: any infectious premise could generate a set of genetic sequences describing possible missing premises. This set of sequences could then be used to compute a new probability of transmission from missing premises, to be compared with the probabilities of transmission from internal and external sources. We leave this for future work.

Other minor assumptions in our model can be readily eased. We hypothesized that all premises have the same infection potential; however, it would be straightforward to make the infectiousness parameter 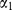 in the model a function of the specific characteristic of the premise, like size or composition (for example, for FMDV sheep are considered to be less infectious than cows, which are in turn less infectious than pigs [17]). Moreover, we note that the infectious potential felt by a premise at time 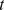 is the sum of the contributions deriving from all the other premises that are infectious at that particular time. As unsampled premises could also contribute to this potential, the temporal dynamics of infection could be modeled in a more complex manner than the step function adopted here. The estimation of the age of an infection from clinical signs is used as a prior distribution in our scheme: an accurate knowledge of this quantity makes the inference computationally more efficient, but it is not essential, and the method can be applied to cases where this quantity is not available. The model used for the mutations of the virus is very simple and does not account for the specific characteristiscs of the FMDV genome, or for some well-known mutation biases (like the transition/transversion bias observed in [20]: we decided once more to go for the simplest and more general assumption, while more detailed and pathogen-specific mutation models could easily be incorporated in our framework.

Our “hosts” do not necessarily correspond to single animals/humans but were interpreted in a wider sense as “infectious units”. These units do not constitute a limitation to our method: even in the case of an infection where the units are individuals, the genetic divergence between sequencing results from an unknown number of viral replications in the donor individual post sampling (but prior to transmission) and in the recipient prior to sampling. In the case of a higher-order unit of infection, the genetic divergence between sequences from sequential samples will be just the result of a larger unknown number of generations.

It is conceivable that multiple pathogen strains circulated on a single premise remained unsampled and went on to infect other premises. For example, FMDV is known to generate independent populations within single animals [20] and different genomes could circulate on a premise. Ideally, several sequences from each premise should be obtained and these data incorporated into the model. Finally, for the specific pathogen considered here, we have used a fixed substitution rate 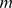 for both the Darlington cluster and the 2007 outbreak. Independent estimates obtained for the whole 2001 epidemic [21] and for 2007 outbreak yield very similar values, which do not change substantially the likelihoods of observing the sequenced genomes. In other applications, the substitution rate may be poorly known. In these cases, it could be viewed as an unknown parameter and estimated in the MCMC simulation.

Computation time is a key element for a method that is expected to be useful in real-time during an outbreak. The computation time was strongly reduced by using a conditional pseudo-distribution of observed sequences 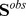 instead of the exact conditional distribution. Clearly, it would be ideal to run the Bayesian estimation using the exact conditional distribution of observed sequences 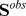. To do so, one could incorporate in the MCMC the unknown transmitted genetic sequences 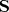 as augmented data (see Eq. (3) below), initialize 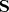 using for example statistical parsimony [16] and determine a proposal distribution for 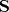 based on a stochastic algorithm estimating genetic networks [22]. Unfortunately, this strategy is at present unfeasible on standard computing resources. However, despite the use of a pseudo-distribution, the running time of our inference algorithm strongly increases with the number of premises. We stress that the main focus of this work was to combine epidemiological and genetic data in a coherent framework, rather than producing an optimised code. Basic optimization procedures should dramatically increase the efficiency of the code. In particular, we suggest three directions worth pursuing: (i) use a conditional pseudo-distribution of the genetic sequences which can be computed faster, but still yielding a good approximation of the posterior distribution of the unknowns; (ii) parallelize the MCMC [23] and code it in a lower-level language; (iii) use alternative algorithms, such as sequential Monte Carlo [24].

Our bayesian inference scheme is a rigorous general platform on which different models can be implemented and tested. It is a useful tool that could be used in real time to detect the presence of missing links in inferred chains of transmission, and to assign confidence values to each inferred transmission event. The specific model we chose for FMDV contains a representation of the dynamics of FMD infections. Different models could be implemented to describe the dynamics of different pathogens, or the specific characteristics of a particular outbreak, while still maintaining rigorous estimation based on genetic and epidemiologic data. Previous work was initiated by Cottam *et al.* [13], and significantly extended by Jombart *et al.* [12] and Ypma *et al.* [14]: all these studies considered the likelihood of the transmission tree 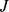 given temporal, spatial and genetic data (here denoted by the generic vectors 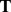, 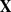 and 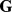) as a product of three independent likelihoods: 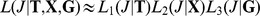. Cottam *et al.* assumed a binary (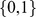) 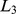 and a uniform 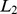 (their estimation does not depend on the location of the premises); Jombart *et al.* designed a less “ad hoc” approach by introducing a maximum parsimony strategy to weight genetic similarity, while spatial and temporal information were considered only when several possible ancestors were genetically indistinguishable; finally Ypma *et al.* had more complicated forms for these likelihood functions. Our method can be considered as the “next step” on this road, as we relax the assumption of independence between the information sources, and we estimate the likelihood of transmission trees given all the sources of information simultaneously. Although some specific aspects of our inference scheme can be refined, expressing the likelihood of a transmission tree as a joint likelihood, depending on both epidemiological and genetic data, significantly advances this form of analysis.

## Materials and Methods

### Data sets

The test data sets analyzed in the Results section were simulated under the model presented below and in Text S1. In these data sets, the outbreak spread over 20 premises (F1, …, F20), randomly and uniformly located in a rectangular 20×10 km region. Values of transmission and latency parameters were 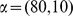 and 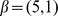. Observed sequences had length 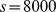 and substitution rate 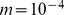. In Text S1, we analyzed an upscaled test data set with 100 premises, with the same premise density as above, and same values for parameters 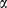, 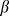, 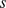 and 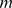.

The data corresponding to the 2007 FMDV outbreak in the UK and to the Darlington cluster within the 2001 epidemic can be found in Refs. [15] and [13], respectively, and are incudedin the Datasets S1, S2, S3, S4. In particular, FMDV sequence length was 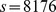 and the substitution rate 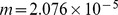 per nt per day [13].

### Observed and unobserved variables

Consider a cluster of 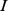 infected hosts (in this case premises) whose centroids are located at Longitude-Latitude coordinates 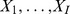. Let 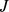 be the function defining the transmission tree: a given premise 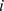 is infected by a source 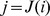, which consists of either another premise 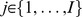, 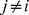, or an external source denoted by 0. For each premise, we consider four timing variables as illustrated by Fig. 1: premise 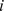 is infected by 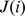 at time 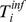, is infectious at time 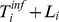, where 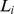 is the latency duration for premise 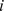, is detected as infected at time 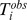 and is removed from the infectious population at time 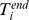. The duration from infectiousness to detection, 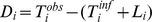, is assessed by experts on the base of clinical signs: let 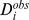 denote this assessment. At time 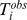, the pathogen is sampled on premise 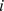 and the genomes are collected for sequencing: let 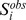 denote the observed consensus sequence.

Among these variables, only 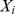, 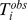, 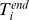, 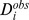 and 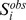 are observed. The others are latent variables to be reconstructed with the bayesian inference scheme.

### Model structure

In this section we briefly describe the essence of the model. The complete specification of the model is provided in the following sections. For a full description of the symbols, we refer to Table 2.

**Table 2:** Description of symbols used in the model.

Symbol	Description
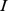	Number of premises in the cluster
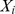	2D–coordinates of the centroid of premise 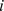
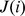	Source of premise 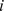 ( 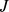 is a function representing the transmission tree)
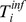	Time of infection of premise 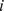 ( 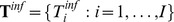 )
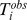	Time of first observation of the pathogen in premise 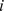 ( 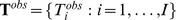 )
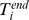	Time of removal of premise 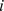 ( 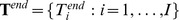 )
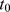	First possible infection time, in this work set to −5
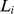	Latency in premise 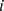 ; 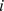 become infectious at time 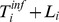 ( 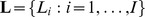 )
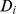	Duration from infectiousness to detection satisfying 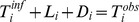 ( 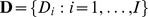 )
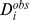	Observed duration from infectiousness to detection ( 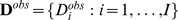 ) (estimated by clinicians based on symptom inspections)
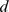	Fixed parameter measuring the uncertainty of 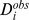 ( 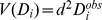 )
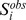	Sequence sampled in premise 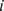 at time 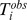 ( 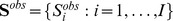 )
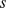	Fixed length of sampled sequences
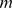	Fixed genetic substitution rate per nucleotide per day
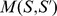	Genetic distance between sequences 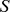 and 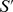
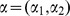	Transmission parameters (source strength and dispersion parameter)
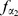	Transmission kernel
	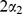 is the mean transmission distance (for an exponential kernel)
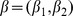	Latency parameters (mean and standard deviation of latency durations, respectively)
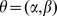	Set of unknown parameters
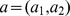	Fixed parameters for the prior distribution of 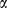
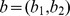	Fixed parameters for the prior distribution of 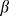

**Figure 5:**
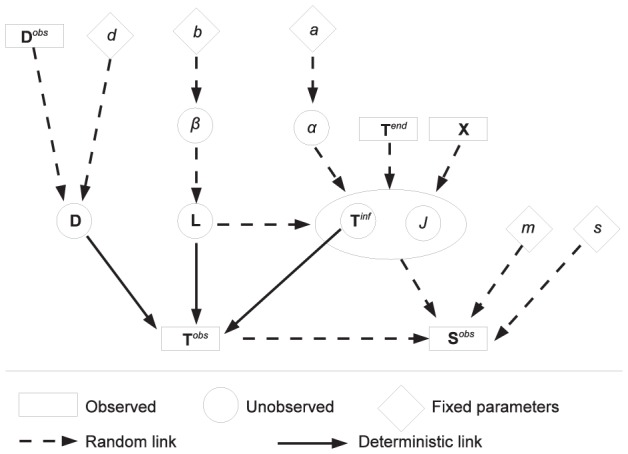
Direct acyclic graph illustrating the dependencies in the model. Bold letters are used to represent sets of variables, with one variable per farm, e.g. 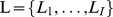. For a full description of the symbols, see Table 2.

Our model for the dynamics of an infection takes into account the dependence between timing, space and genetics. It includes (i) the delays between infection and observation of infection and (ii) the difference between transmitted and observed genetic sequences of the pathogen. The direct acyclic graph (DAG) in Fig. 5 shows the structure of the model. Upper case letters are used for latent and observed variables, while Greek letters denote unknown parameters. Lower case letters are used for fixed parameters. Observation times 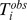 and observed consensus sequences 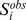 are viewed as response variables. They depend on the transmission tree and on the temporal dynamics (infection times, latency durations and detection durations).

The model assumes that the epidemic starts with the infection of a single premise from an external source. Then, transmissions 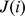 and infection times 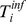 depend on the infection potential generated by previously infected premises. The infection potential depends on the transmission parameters 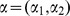, the spatial location of premises and the times at which infected premises exit from latency and are removed from the infectious population: an infected premise 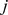 is infectious between 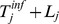 and 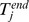, and the probability of infecting premise 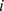 decreases exponentially with the distance 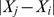. The parameter 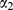 appears in the transmission kernel 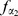 and quantifies the decrease with distance of the infection potential of each infectious premise, while 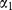 quantifies the infection strength of each infectious premise. The mean transmission length, defined here as 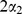, is a function of the distances between farms and of the transmission kernel we used. Latency durations 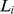 and durations from infectiousness to the time that virus is sampled 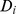 are assumed to be independent. The distribution of 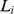 is parametrised by its expectation 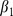 and its variance 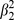; 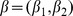 is the vector of latency parameters. The distribution of 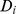 is centered around the empirical estimate 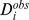 but has a variance increasing with 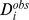, equal to 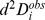, where 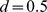. The premise index 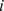 is sorted with respect to increasing infection times 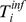.

### Posterior distribution

We aim to assess the joint posterior distribution 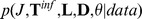 of the transmission tree 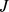, infection times 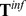, latency durations 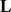, durations from infectiousness to detection 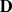, and parameters 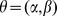, given the data. Data are observed sequences 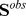, pathogen observation times 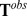, observed durations from infectiousness to detection 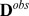, removal times 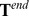 and premise locations 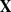:
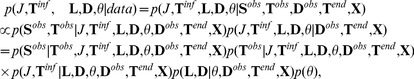
(1)where 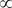 means “proportional to” (the multiplicative constant does not depend on the unknowns 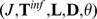). In this decomposition, 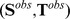 are viewed as response variables (or model output), 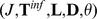 as latent variables and 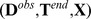 as explanatory variables. The term 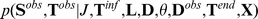 is the complete likelihood of the model and the term 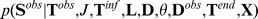 is the conditional complete likelihood of the model given observation times 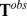. In the following sections, we specify the terms appearing in the last two lines of Equation (1).

### Conditional distribution of observed sequences 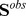


Assumptions: (a) there is only one sequence per infected premise; (b) sequences in all the premises evolve at a constant rate 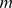 (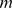 is the substitution rate per day per nucleotide).

The model for 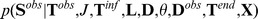 is based on the probability distribution of the number of substitutions between two sequences during the evolutionary durations separating the sequences. Let 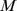 denote the number of substitutions and 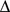 the evolutionary duration (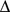 is the sum of time intervals computed along the transmission tree). The conditional distribution of 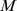 given 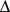 is a Binomial distribution taking into account the Jukes-Cantor's correction (see Text S1):
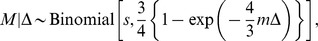
and the probability of 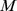 given 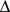 is:
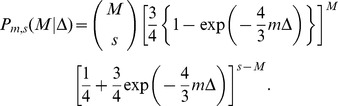
(2)


Therefore, 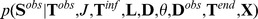 does not depend on 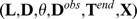:
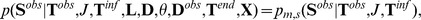
and can be written as a multiple sum of products of binomial probabilities. The sum is computed over the unknown transmitted genetic sequences, say 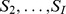, at time 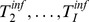 (the initial sequence of the root 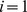 of the tree is not needed):
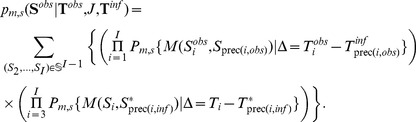
(3)In Equation (3), 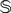 is the set of all possible sequences (the size of 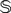 is 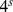, where 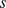 is the length of the sequence); 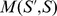 is the number of substitutions between 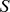 and 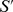; 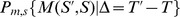 is the probability given by Equation (2) with 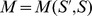 and 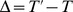. The subscript 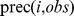 denotes the premise whose node of infection belongs to the tree path from the root of the tree to the observation of 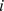 (at time 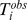) and whose infection is just preceding the observation of 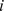. The node of infection of a given premise 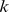 is defined as the point on the tree at which “the branch leading to the observation of 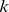
” and “the branch leading to the observation of the infecting premise 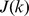
” diverged. The tree path from one point of the tree to another is defined as the most direct path on the graph conncting the two points. If 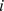 did not infect any other premise, then 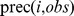 is 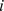 itself. In the particular case where 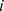 was infected after the observation of the infecting farm 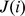 and 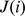 did not infect any other premise between 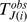 and 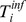
”, the subscript 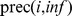 coincides with 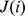, 
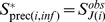
 and 
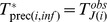
. In the most frequent other cases, 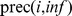 denotes the premise whose node of infection belongs to the tree path from the root of the tree to the infection of 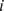 (at time 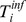) and whose infection is just preceding the infection of 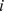; in these cases, 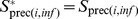 and 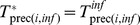. In other words, the first series of factors in Equation (3) accounts for the probabilities of the number of substitutions between an observed sequence and the immediately preceding unobserved, transmitted sequence, while the second series of factors accounts for the probabilities of the number of substitutions between each transmitted sequence and the transmitted or observed sequences immediately preceding in time. Equation (3) is written in the Supporting Text S1 (Equation (2)) for the simple transmission tree drawn in Supporting Fig. S1.

### Conditional pseudo-distribution of observed sequences 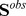


The conditional distribution for 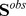 (Eq. (3)) was written as a distribution depending solely on the genetic distances 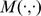 for pairs of sequences. However, in each pair, there is at least one unobserved transmitted sequence. Therefore, exploiting Equation (3) would lead us to consider extra latent variables (or augmented data), namely the unobserved sequences 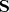. In order to reduce the complexity of the posterior, we preferred not to include these extra latent variables, but rather to use a conditional pseudo-distribution of 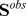, 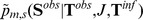. In our method, 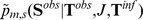 replaces 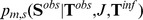 which is the conditional complete likelihood of the model given observation times 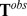. Thus, 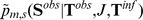 is a conditional complete pseudo-likelihood given observation times and we refer to it as a conditional pseudo-distribution. It follows that the posterior distribution that we assess is actually a pseudo-posterior distribution.

With index 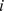 being sorted with respect to increasing infection times 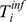, 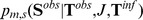 can be written:
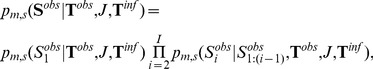
(4)where 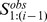 is the set of observed sequences of premise 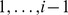. We considered the sequence 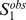 of the first infected premise as arbitrary. Thus, 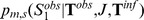 was discarded in the pseudo-distribution. Moreover, to compute exactly 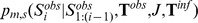 appearing in Equation (4), we should write this probability as a sum over the unknown transmitted genetic sequences (as done in Equation (3)). In order to avoid the inclusion of unknown transmitted sequences as augmented data, we replaced, for 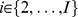, the conditional probability 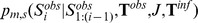 of 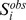 given past sequences 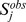 (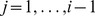) by the product of the conditional probabilities of 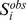 given *each* past sequence 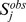 (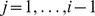):
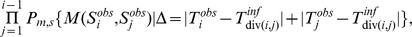
where 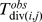 denotes the infection time at which the chain of infection leading to 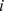 and the chain of infection leading to 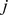 diverged (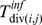 is one of the latent variables in 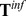, also called “augmented data”) and 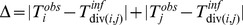 is the evolutionary duration separating the observation of 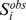 and 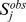. Thus, the conditional pseudo-distribution of 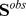 satisfies:
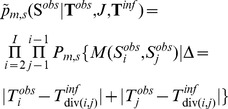
(5)The right hand side of Equation (5) replaces 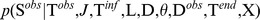 in Equation (1). Equation (5) is written in Equation (3) in Text S1 for the simple transmission tree drawn in Fig. S1.

We tested another form for 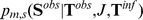, described in Text S1. The form given by Equation (5) above led to the best reconstruction of the transmission tree 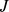.

### Conditional distribution of pathogen observation times 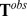



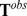
 satisfies the relation 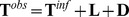. Therefore, the conditional distribution of 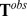 is simply:
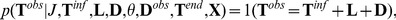
(6)where **1** 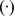 is the indicator function (**1** 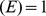 if event 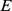 occurs, zero otherwise).

### Joint distribution of transmissions 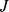 and infection times 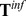


Assumptions: (a) Only one premise is infected by an external source, while the others premises in the dataset are infected by previously-infected premises within the dataset; (b) any premise 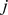 may infect other premises after the latency period 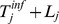 and before the culling time 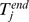; (c) infectious premises have same infection strength 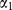, considered constant; (d) the infection risk of a susceptible premise by an infectious premise decreases exponentially with the distance separating both premises, this distance being measured by the distance between the centroids of the premises; (e) the presence of unsampled premises in the area (premises for which genetic or epidemiological data is not available) is ignored.

With the index 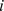 being sorted with respect to increasing infection times 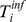, the probability 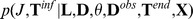 can be written:
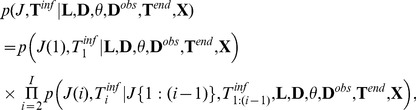
(7)where 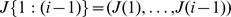 and 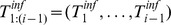.

Each premise has the same chance (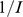) to be infected first (by an external source 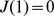), and its infection time is assumed to be greater or equal than a minimum infection time 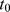 (in this work we used 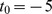), and less than or equal to the minimum removal time 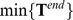:
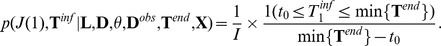
Subsequent infections occur with the following probabilities:
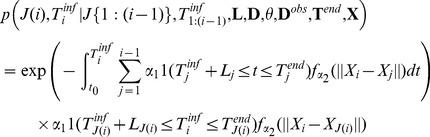
where the term 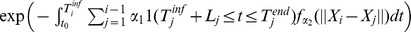 is the probability that premise 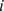 has not been infected until time 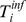 by the previously infected premises 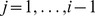, and the term 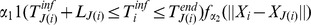 is the probability density that premise 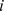 has been infected by 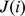 at time 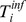. The function 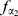 is an exponential transmission kernel, defined for all distance 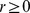 as
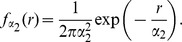
For transmissions modelled using the exponential transmission kernel, the mean transmission distance (mean length of transmissions) is 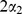: this measure depends on the distances between farms as well as on the transmission kernel we used. Other transmission kernels, such as those presented in [25], [26] could be tested. The selection of the best transmission kernel will be crucial for datasets with large number of premises and large spatial extent. In our applications, where the number of premises is limited and the spatial extent is much smaller than the dispersal capacity of the pathogen, there are enough data to infer the transmission parameters, but not enough to carry out a significant model selection about the transmission kernel.

### Distributions of latency durations 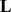 and detection durations 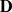


Assumptions: (a) a priori, latencies and durations from infectiousness to detection are independent; (b) characteristics of the latency distribution (expectation and variance) do not depend on time and premise; (c) the expectation (resp. variance) of the duration from infectiousness to observation is equal to (resp. is proportional to) the estimate provided.

We chose gamma distributions for latency durations 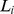, with shape and scale parameters 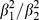 and 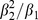, respectively, so that 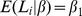 and 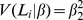. We refer to 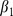 as mean latency duration. We chose gamma distributions for detection durations 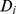 with shape and scale parameters 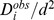 and 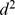, respectively, so that 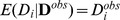 and 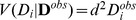. Thus, the joint distribution of the vectors of latent variables 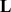 and 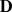 satisfies:
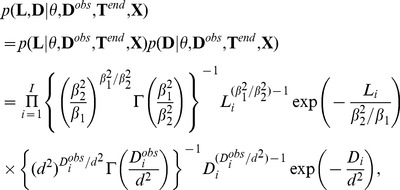
(8)where 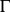 is the gamma function.

### Prior distribution of parameters 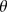


The four components of 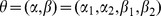 have independent exponential priors with mean parameters 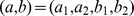:
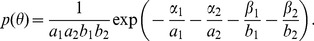
(9)We have used the values 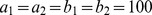.

### Bayesian inference

We built a Monte Carlo Markov Chain (MCMC) algorithm to assess the posterior distribution of 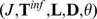, coded in the R language [27]. Details of this algorithm are provided in Text S1. We recall that, in order to reduce the complexity of the algorithm, we replaced the conditional distribution of observed consensus sequences appearing in the posterior distribution by a pseudo-distribution. This replacement allowed us to remove some of the latent variables, namely the unobserved pathogen sequences transmitted at the infection times. Therefore, the MCMC algorithm assesses a pseudo-posterior distribution of 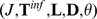. Vague priors were used for parameters 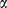 and 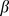 (see above). In the cases considered in this study, 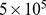 iterations of the MCMC algorithm were enough to assess the posterior distributions of the unknowns. Running 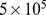 iterations took about two days for the simulation with 20 premises and one month for the simulation with 100 premises on an Intel Xeon Quad Core processor with clock speed 2.93 GHz and 48 Gb of RAM memory. The components of the algorithm which are especially computationally costly are (i) the search of the most recent ancestral premises appearing in the pseudo-distribution of the observed genetic sequences given in Equation (5), (ii) the computation of the joint distribution of 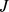 and 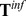 in Equation (7) which is based on a convolution between the transmission kernel and the sources of infection, and (iii) the verification that timing constraints are satisfied when infection times are updated (see proposal distributions in Text S1).

### Simulation datasets to assess the performance of the inference

We generated data sets using the model described above and the location of the premises. The spread of the disease was first simulated using the conditional distributions of 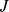, 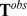, 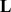, 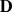, 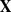 and 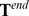, with previously inferred parameters, thus obtaining the complete dynamics of the infection and a transmission tree. Subsequently, genetic distances between the observed sequences were generated using the binomial distributions described in Equation (2). We note that in this case we generated the unobserved transmitted sequences as well.

## Supporting Information

Data S1 **FMDV complete genomes for the 2007 dataset.**
(FASTA)

Data S2 **Epidemiological data for the 2007 dataset.**
(TXT)

Data S3 **FMDV complete genomes for the 2001 dataset.**
(FASTA)

Data S4 **Epidemiological data for the 2001 dataset.**
(TXT)

Figure S1
**Example of transmissions between four spatially-confined premises** 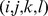 **.** Bold lines: time intervals 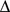 appearing in Equation (2) in Text S1, over which the true conditional distributions of observed sequences can be computed.(TIF)

Figure S2 **Simulated outbreak.** Trees with the five highest posterior probabilities (coloured disks) and true transmissions (black circles).
(TIF)

Figure S3
**Simulated outbreak.** Posterior distributions (histograms) of parameters. Top four panels: parameters 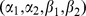; dashed line: true value; dotted-dashed curve: prior distribution; solid line: posterior median; dotted lines: posterior quantiles 0.025 and 0.975. Bottom left: transmission kernel, depending on parameter 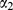; dashed curve: true kernel; solid curve: posterior median; dotted-dashed curves: posterior quartiles 0.25 and 0.75; dotted curves: posterior quantile 0.025 and 0.975. Bottom center: posterior sample of 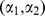 provided by the MCMC, showing a strong dependence in the joint posterior distribution. Bottom right: posterior sample of 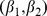 provided by the MCMC.(TIF)

Figure S4 **Simulated outbreak.** Posterior distributions of infection times (top) and latency durations (bottom left) for the simulated outbreak. In both panels, vertical solid lines indicate the true values. In the top panel, vertical dashed lines indicate the virus observation times.
(TIF)

Figure S5 **2007 UK epidemics.** Trees with the five highest posterior probabilities (coloured disks).
(TIF)

Figure S6
**2007 UK epidemics.** Posterior distributions (histograms) of parameters. Top four panels: 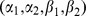; dotted-dashed curve: prior distribution; solid line: posterior median; dotted lines: posterior quantiles 0.025 and 0.975. Bottom left: transmission kernel, depending on parameter 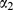; solid curve: posterior median; dotted-dashed curves: posterior quartiles 0.25 and 0.75; dotted curves: posterior quantile 0.025 and 0.975. Bottom center: posterior sample of 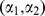 provided by the MCMC, showing a strong dependence in the joint posterior distribution. Bottom right: posterior sample of 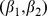 provided by the MCMC.(TIF)

Figure S7 **2007 UK epidemics.** Posterior distributions of infection times (top) and latency durations (bottom left). In the top panel, vertical dashed lines indicate the virus observation times.
(TIF)

Figure S8 **2001 epidemics, Darlington cluster including premise B.** Top left: Posterior probabilities of transmissions (dots with varying size). Top right: Tree with the highest posterior probability mapped in space (arrows). Bottom: Trees with the five highest posterior probabilities (coloured disks).
(TIF)

Figure S9 **2001 epidemics, Darlington cluster without premise B.** Trees with the highest posterior probabilities (coloured disks).
(TIF)

Figure S10
**2001 epidemics, Darlington cluster without premise B.** Posterior distributions (histograms) of parameters. Top four panels: 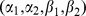; dotted-dashed curve: prior distribution; solid line: posterior median; dotted lines: posterior quantiles 0.025 and 0.975. Bottom left: transmission kernel which depends on parameter 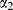; solid curve: posterior median; dotted-dashed curves: posterior quartiles 0.25 and 0.75; dotted curves: posterior quantile 0.025 and 0.975. Bottom center: posterior sample of 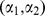 provided by the MCMC, showing a strong dependence in the joint posterior distribution. Bottom right: posterior sample of 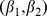 provided by the MCMC.(TIF)

Figure S11 **2001 epidemics, Darlington cluster without premise B.** Posterior distributions of infection times (top) and latency durations (bottom left). In the top panel, vertical dashed lines indicate the virus observation times.
(TIF)

Figure S12
**Spatial representation of the tree with the highest posterior probability, for different parametrisations of the prior distribution for the veterinarian assessment of the age of the oldest lesion on a premise.** Left column: 2007 epidemics, right column: cluster in the 2001 epidemics. Top: prior variance of 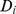 equal to 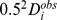. Center: prior variance of 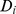 set to 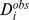 (information provided by the veterinarians are more uncertain). Bottom: prior variance of 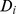 set to 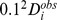 (information provided by the veterinarians are less uncertain). See also Fig. S13.(TIF)

Figure S13
**Uncertainty about the veterinarian assessment of the age of the oldest lesion on a premise, for different parametrisations of the prior distribution.** Left: prior variance of 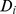 set to 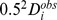. Center: prior variance of 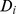 set to 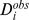 (information provided by the veterinarians are more uncertain). Right: prior variance of 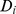 set to 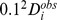 (information provided by the veterinarians are less uncertain).(TIF)

Figure S14 **Genetic network, based on statistical parsimony, implemented in the software package TCS** [**16**] **.** Full dots represent observed genomes, while empty dots represent unsampled genomes (for these last ones, timing is arbitrary), links represent single mutations. Top panel: subset of the Darlington cluster, 2001 UK FMDV epidemics [13]; bottom panel: 2007 UK FMDV epidemics [15]. Each arrow indicates the network root, based on the references above.
(TIF)

Figure S15 **Transmissions for the simulated outbreak with 100 farms.** True transmissions are indicated with circles; dot sizes are proportional to posterior probabilities of transmissions.
(TIF)

Figure S16 **Tree with the highest posterior probablity - simulated outbreak with 100 farms.** The tree has been divided in 4 panels (premises 1–25, 26–50, 51–75, 76–100 respectively) for clarity. Solid arrows represent inferred transmissions. When the inference is not correct, the true transmission is drawn as a dotted-dashed arrow.
(TIF)

Figure S17
**Posterior distributions (histograms) - simulated outbreak with 100 farms.** Posterior distributions of mean latency duration (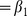; left) and mean transmission distance (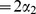; right); dashed lines: true values; dotted-dashed curves: prior distributions; solid lines: posterior medians; dotted lines: posterior quantiles 0.025 and 0.975.(TIF)

Table S1 **Additional criteria to assess the performance of the estimation algorithm over three series of 100 simulations (test, 2007, 2001).** Criteria are the coverages by the 95% posterior intervals of the infection times, the times at which the premises became infectious, the transmission parameters (source strength and dispersion parameter) and the latency parameters (mean and Sd.).
(PDF)

Text S1 **Details about the mathematical model, the Monte Carlo Markov Chain Algorithm, further analyses of its performances and comparison with previous results in the literature.**
(PDF)
