# Supplementary figures and images for: A Bayesian Inference Framework to Reconstruct Transmission Trees Using Epidemiological and Genetic Data
Source: PLoS Comput Biol. 2012 Nov 15;8(11):e1002768. doi: 10.1371/journal.pcbi.1002768 (PMC3499255; doi:10.1371/journal.pcbi.1002768)

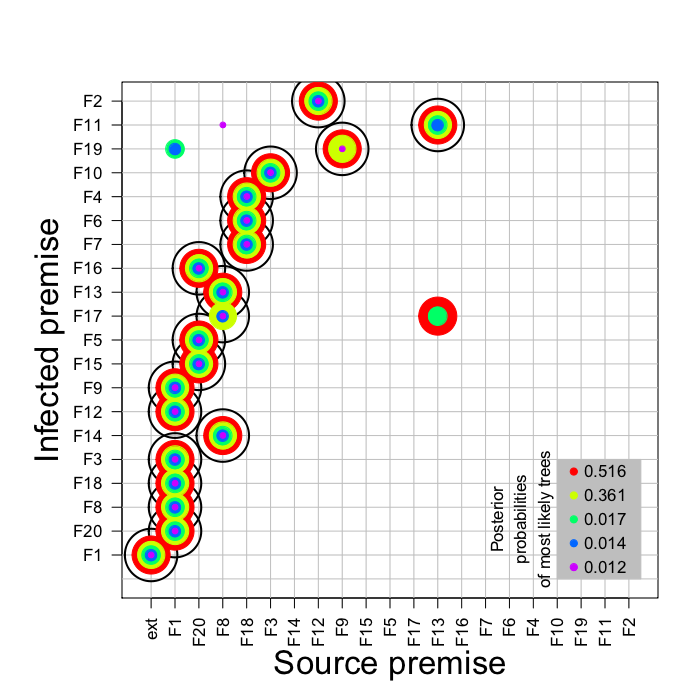

Supplement: Figure S2 — Simulated outbreak. Trees with the five highest posterior probabilities (coloured disks) and true transmissions (black circles). (TIF) [file pcbi.1002768.s006.tif]

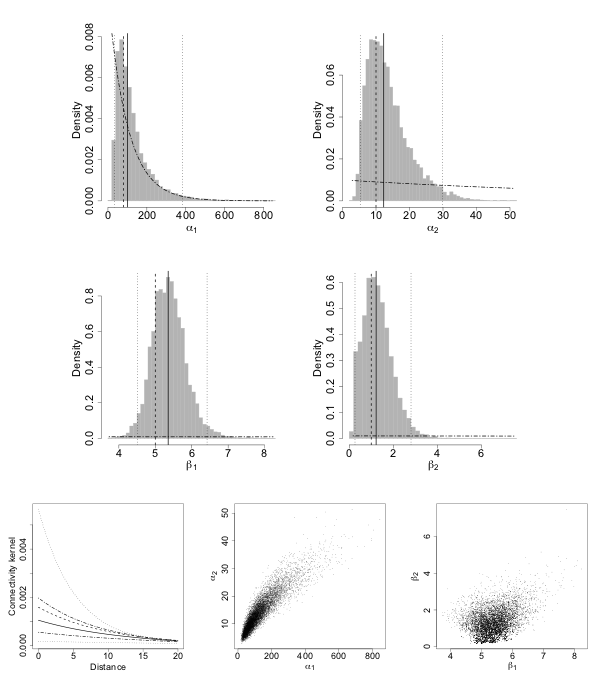

Supplement: Figure S3 — Simulated outbreak. Posterior distributions (histograms) of parameters. Top four panels: parameters ; dashed line: true value; dotted-dashed curve: prior distribution; solid line: posterior median; dotted lines: posterior quantiles 0.025 and 0.975. Bottom left: transmission kernel, depending on parameter ; dashed curve: true kernel; solid curve: posterior median; dotted-dashed curves: posterior quartiles 0.25 and 0.75; dotted curves: posterior quantile 0.025 and 0.975. Bottom center: posterior sample of provided by the MCMC, showing a strong dependence in the joint posterior distribution. Bottom right: posterior sample of provided by the MCMC. (TIF) [file pcbi.1002768.s007.tif]

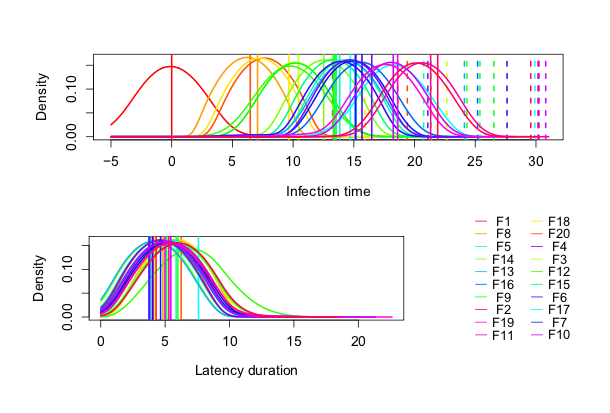

Supplement: Figure S4 — Simulated outbreak. Posterior distributions of infection times (top) and latency durations (bottom left) for the simulated outbreak. In both panels, vertical solid lines indicate the true values. In the top panel, vertical dashed lines indicate the virus observation times. (TIF) [file pcbi.1002768.s008.tif]

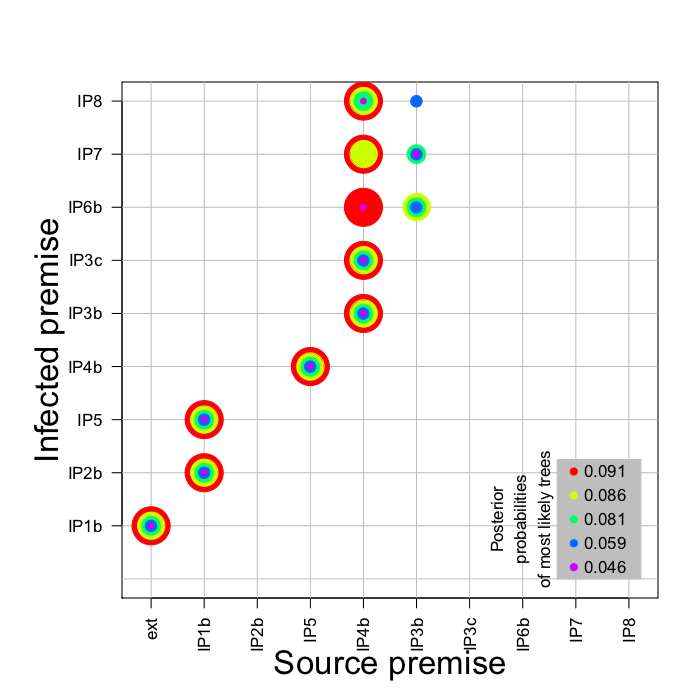

Supplement: Figure S5 — 2007 UK epidemics. Trees with the five highest posterior probabilities (coloured disks). (TIF) [file pcbi.1002768.s009.tif]

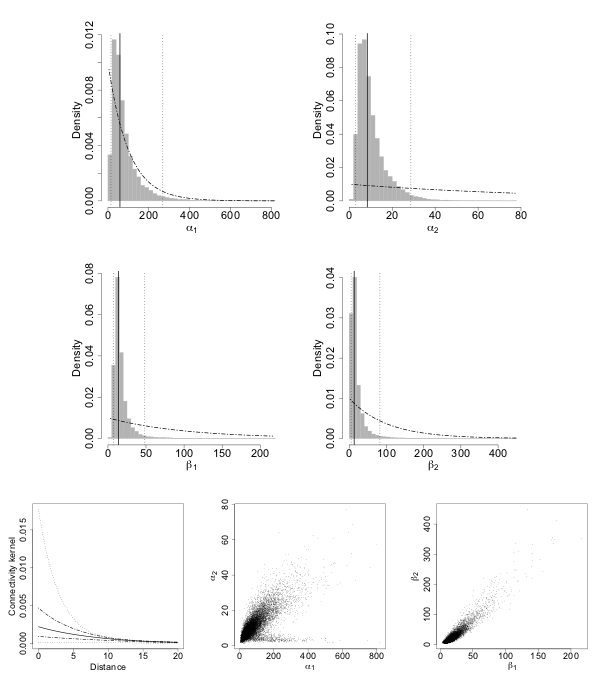

Supplement: Figure S6 — 2007 UK epidemics. Posterior distributions (histograms) of parameters. Top four panels: ; dotted-dashed curve: prior distribution; solid line: posterior median; dotted lines: posterior quantiles 0.025 and 0.975. Bottom left: transmission kernel, depending on parameter ; solid curve: posterior median; dotted-dashed curves: posterior quartiles 0.25 and 0.75; dotted curves: posterior quantile 0.025 and 0.975. Bottom center: posterior sample of provided by the MCMC, showing a strong dependence in the joint posterior distribution. Bottom right: posterior sample of provided by the MCMC. (TIF) [file pcbi.1002768.s010.tif]

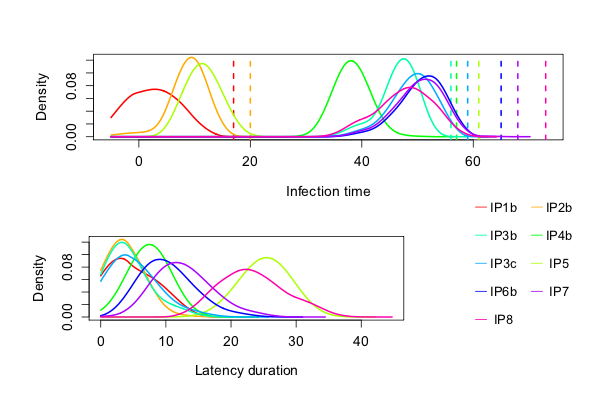

Supplement: Figure S7 — 2007 UK epidemics. Posterior distributions of infection times (top) and latency durations (bottom left). In the top panel, vertical dashed lines indicate the virus observation times. (TIF) [file pcbi.1002768.s011.tif]

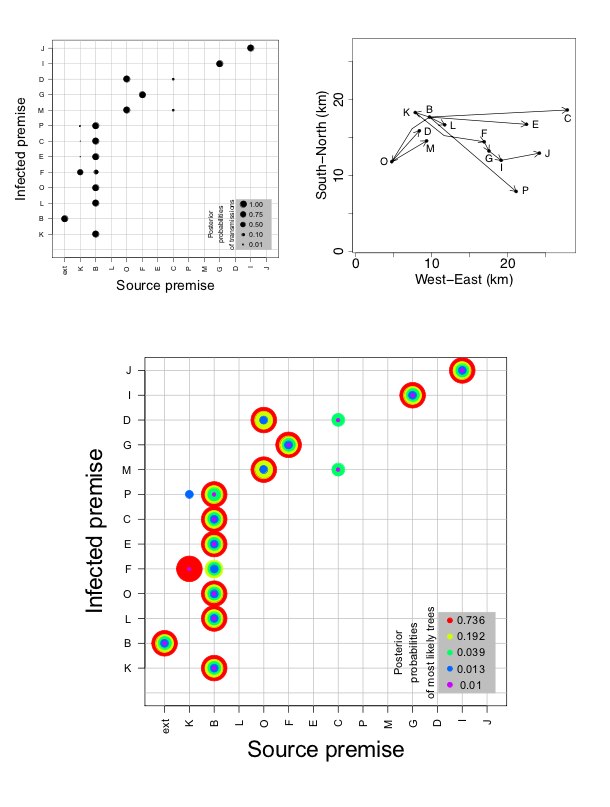

Supplement: Figure S8 — 2001 epidemics, Darlington cluster including premise B. Top left: Posterior probabilities of transmissions (dots with varying size). Top right: Tree with the highest posterior probability mapped in space (arrows). Bottom: Trees with the five highest posterior probabilities (coloured disks). (TIF) [file pcbi.1002768.s012.tif]

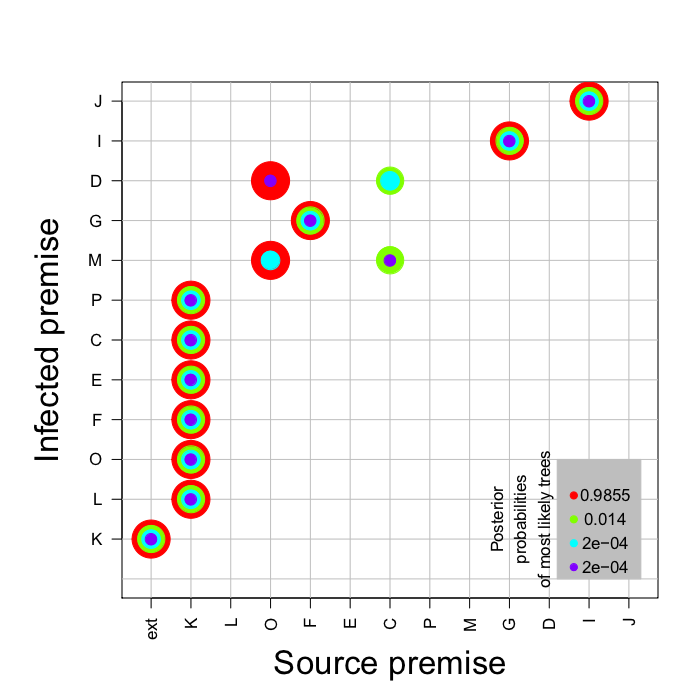

Supplement: Figure S9 — 2001 epidemics, Darlington cluster without premise B. Trees with the highest posterior probabilities (coloured disks). (TIF) [file pcbi.1002768.s013.tif]

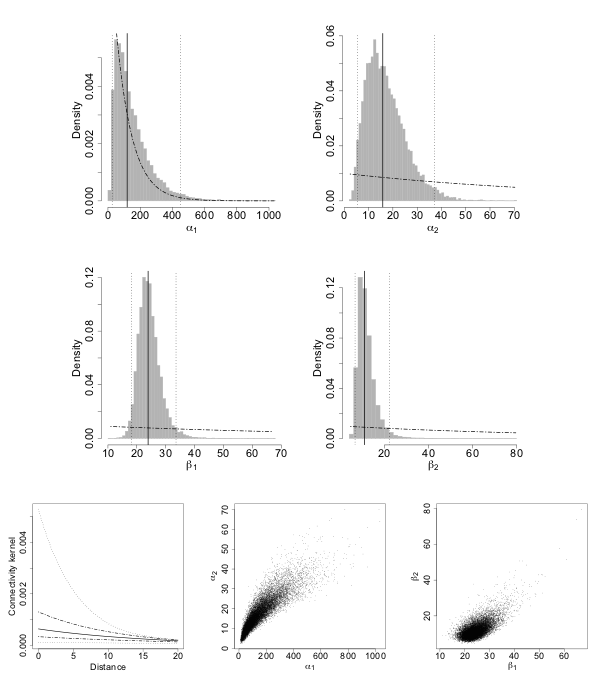

Supplement: Figure S10 — 2001 epidemics, Darlington cluster without premise B. Posterior distributions (histograms) of parameters. Top four panels: ; dotted-dashed curve: prior distribution; solid line: posterior median; dotted lines: posterior quantiles 0.025 and 0.975. Bottom left: transmission kernel which depends on parameter ; solid curve: posterior median; dotted-dashed curves: posterior quartiles 0.25 and 0.75; dotted curves: posterior quantile 0.025 and 0.975. Bottom center: posterior sample of provided by the MCMC, showing a strong dependence in the joint posterior distribution. Bottom right: posterior sample of provided by the MCMC. (TIF) [file pcbi.1002768.s014.tif]

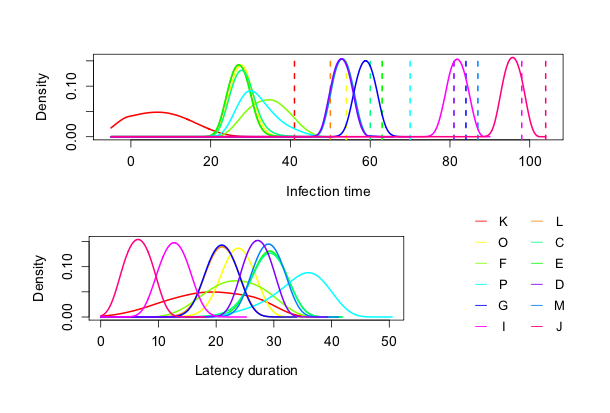

Supplement: Figure S11 — 2001 epidemics, Darlington cluster without premise B. Posterior distributions of infection times (top) and latency durations (bottom left). In the top panel, vertical dashed lines indicate the virus observation times. (TIF) [file pcbi.1002768.s015.tif]

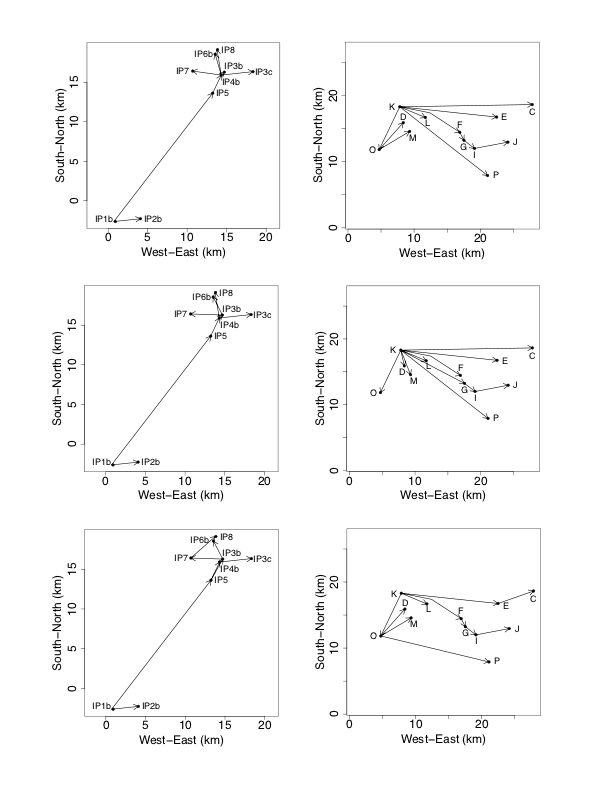

Supplement: Figure S12 — Spatial representation of the tree with the highest posterior probability, for different parametrisations of the prior distribution for the veterinarian assessment of the age of the oldest lesion on a premise. Left column: 2007 epidemics, right column: cluster in the 2001 epidemics. Top: prior variance of equal to . Center: prior variance of set to (information provided by the veterinarians are more uncertain). Bottom: prior variance of set to (information provided by the veterinarians are less uncertain). See also Fig. S13. (TIF) [file pcbi.1002768.s016.tif]

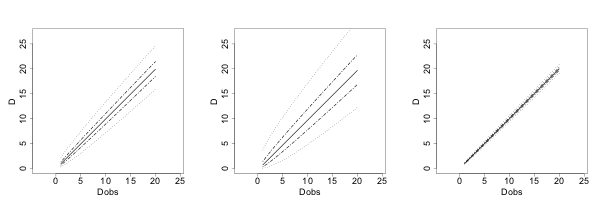

Supplement: Figure S13 — Uncertainty about the veterinarian assessment of the age of the oldest lesion on a premise, for different parametrisations of the prior distribution. Left: prior variance of set to . Center: prior variance of set to (information provided by the veterinarians are more uncertain). Right: prior variance of set to (information provided by the veterinarians are less uncertain). (TIF) [file pcbi.1002768.s017.tif]

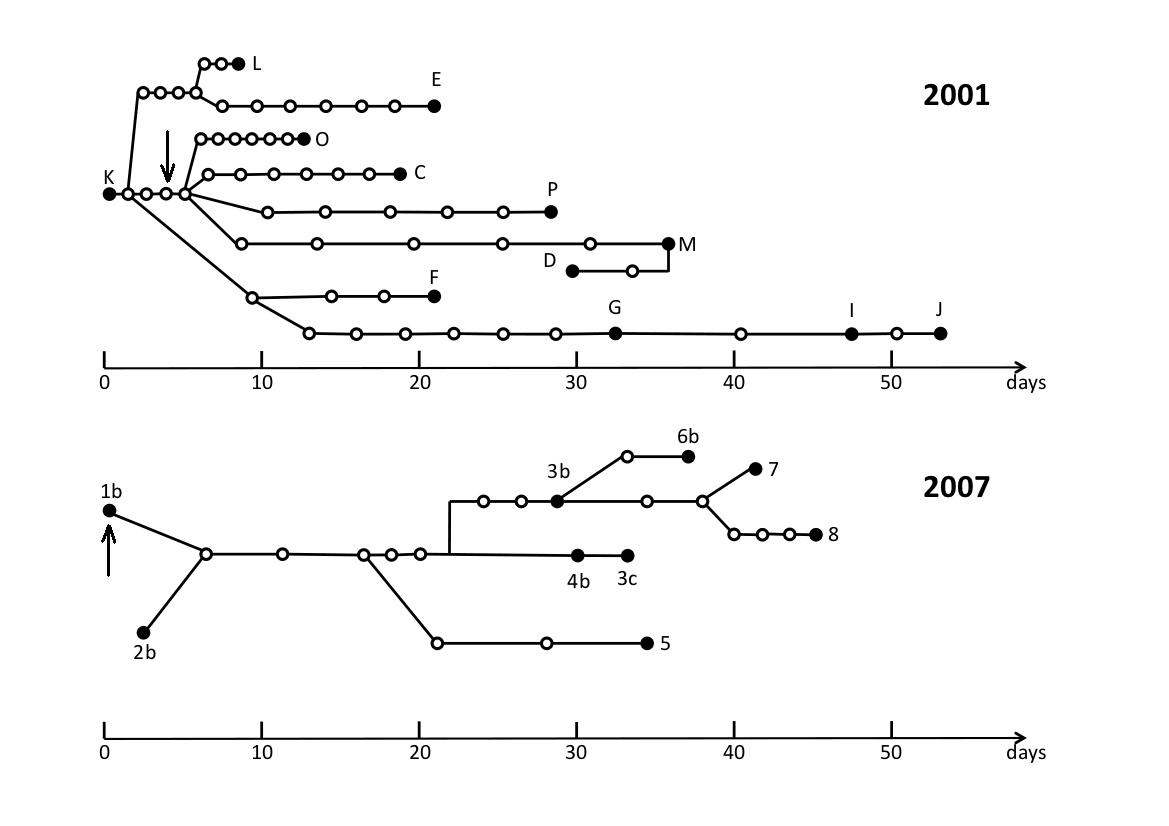

Supplement: Figure S14 — Genetic network, based on statistical parsimony, implemented in the software package TCS [16] . Full dots represent observed genomes, while empty dots represent unsampled genomes (for these last ones, timing is arbitrary), links represent single mutations. Top panel: subset of the Darlington cluster, 2001 UK FMDV epidemics [13]; bottom panel: 2007 UK FMDV epidemics [15]. Each arrow indicates the network root, based on the references above. (TIF) [file pcbi.1002768.s018.tif]
