# Supplementary material for: A Bayesian Inference Framework to Reconstruct Transmission Trees Using Epidemiological and Genetic Data
Source: PLoS Comput Biol. 2012 Nov 15;8(11):e1002768. doi: 10.1371/journal.pcbi.1002768 (PMC3499255; doi:10.1371/journal.pcbi.1002768)
Supplement: Table S1 — Additional criteria to assess the performance of the estimation algorithm over three series of 100 simulations (test, 2007, 2001). Criteria are the coverages by the 95% posterior intervals of the infection times, the times at which the premises became infectious, the transmission parameters (source strength and dispersion parameter) and the latency parameters (mean and Sd.). (PDF) [file pcbi.1002768.s022.pdf]

Coverage	Test	2007	2001
Infection times	0.78	0.81	0.88
Infectiousness times	0.93	0.95	0.93
Source strength	0.97	0.55	1.00
Dispersion parameter	0.89	0.60	1.00
Latency mean	0.94	0.78	1.00
Latency Sd.	0.43	0.83	0.97
