# Supplementary material for: A Bayesian Inference Framework to Reconstruct Transmission Trees Using Epidemiological and Genetic Data
Source: PLoS Comput Biol. 2012 Nov 15;8(11):e1002768. doi: 10.1371/journal.pcbi.1002768 (PMC3499255; doi:10.1371/journal.pcbi.1002768)
Supplement: Text S1 — Details about the mathematical model, the Monte Carlo Markov Chain Algorithm, further analyses of its performances and comparison with previous results in the literature. (PDF) [file pcbi.1002768.s023.pdf]

Supporting text

Model construction

Conditional distribution of the number of substitutions

The model for $p(\mathbf{S}^{obs} \mid \mathbf{T}^{obs}, J, \mathbf{T}^{inf}, \mathbf{L}, \mathbf{D}, \theta, \mathbf{D}^{obs}, \mathbf{T}^{end}, \mathbf{X})$ is based on the probability distribution of the number of substitutions between sequences during the evolutionary durations separating the sequences. Here we derive this probability distribution.

The evolutionary duration Δ between two sequences is the sum of time intervals computed along the transmission tree. For sequences with four modalities per site (A, C, T, G), we assume a constant probability of 1/3 for each possible mutation. We suppose that mutations at one position appear randomly and independently in time, so that the number of mutations during a time interval Δ is a Poisson distribution with intensity $m\Delta$. Let P_Δ be the probability that, at position x, the value at time Δ is the same as the value at time 0. Then, taking into account the Jukes-Cantor's correction,

$$\begin{aligned}
P_{\Delta+d\Delta} = \; & P_\Delta P(\text{no mutation between } \Delta \text{ and } \Delta + d\Delta) \\
+ \; & (1 - P_\Delta)P(\text{one mutation between } \Delta \text{ and } \Delta + d\Delta \text{ going back to the original value}) \\
= \; & P_\Delta(1 - m d\Delta) + (1 - P_\Delta) m d\Delta \tfrac{1}{3}
\end{aligned}$$

Thus $P'_\Delta = (1 - 4P_\Delta)\frac{m}{3}$, and solving this equation with the initial condition $P_0 = 1$ one gets

$$P_\Delta = \frac{1 + 3e^{-\frac{4}{3}m\Delta}}{4}.$$

If a mutation appears, it is uniform between all possible new values, so the probability to observe a given value different from the value at $\Delta = 0$ is $\frac{1-P_\Delta}{3}$. Therefore, the conditional distribution of the number of differences between two sequences given Δ is:

$$M \mid \Delta \sim \text{Binomial}\left[s, \frac{3}{4}\left\{1 - \exp\left(-\frac{4}{3}m\Delta\right)\right\}\right]$$

whose probability is

$$P_{m,s}(M \mid \Delta) = \binom{M}{s} \left[\frac{3}{4}\left\{1 - \exp\left(-\frac{4}{3}m\Delta\right)\right\}\right]^M \left[\frac{1}{4} + \frac{3}{4}\exp\left(-\frac{4}{3}m\Delta\right)\right]^{s-M}. \quad (1)$$

Conditional distribution of observed genetic sequences for a simple tree

For the simple transmission tree drawn in Fig. S1, Eq. (3) in the main text becomes:

$$\sum_{S_k\in\mathbb{S}}\sum_{S_i\in\mathbb{S}}\sum_{S_l\in\mathbb{S}} P_{m,s}\{M(S_k^{obs},S_k)\mid \Delta=T_k^{obs}-T_k^{inf}\}P_{m,s}\{M(S_j^{obs},S_k)\mid \Delta=T_j^{obs}-T_k^{inf}\}$$
$$\times P_{m,s}\{M(S_k,S_i)\mid \Delta=T_k^{inf}-T_i^{inf}\}P_{m,s}\{M(S_l,S_i)\mid \Delta=T_l^{inf}-T_i^{inf}\}$$
$$\times P_{m,s}\{M(S_i^{obs},S_l)\mid \Delta=T_i^{obs}-T_l^{inf}\}P_{m,s}\{M(S_l^{obs},S_l)\mid \Delta=T_l^{obs}-T_l^{inf}\},\quad (2)$$

where S_k, S_i and S_l are genetic sequences transmitted at times T_k^{inf}, T_i^{inf} and T_l^{inf} to premises k, i and l, respectively; $\mathbb{S}$ is the set of all possible sequences (the size of $\mathbb{S}$ is 4^s, where s is the length of the sequence); $M(S',S)$ is the number of substitutions between S and S'; $P_{m,s}\{M(S',S)\mid \Delta=T'-T\}$ is the probability given by Eq. (1) in this document with $M=M(S',S)$ and $\Delta=T'-T$.

Conditional pseudo-distribution of observed genetic sequences for a simple tree

For the simple transmission tree drawn in Fig. S1, $\text{div}(i,j)=i$, $\text{div}(k,j)=k$, $\text{div}(k,i)=i$, $\text{div}(l,j)=i$, $\text{div}(l,i)=l$ and $\text{div}(l,k)=i$ and the conditional pseudo-distribution of observed genetic sequences is:

$$\tilde{p}_{m,s}(\mathbf{S}^{obs}\mid \mathbf{T}^{obs},J,\mathbf{T}^{inf}) = P_{m,s}\{M(S_i^{obs},S_j^{obs})\mid \Delta=\mid T_i^{obs}-T_i^{inf}\mid+\mid T_j^{obs}-T_i^{inf}\mid\}$$
$$\times P_{m,s}\{M(S_k^{obs},S_j^{obs})\mid \Delta=\mid T_k^{obs}-T_k^{inf}\mid+\mid T_j^{obs}-T_k^{inf}\mid\}$$
$$\times P_{m,s}\{M(S_k^{obs},S_i^{obs})\mid \Delta=\mid T_k^{obs}-T_i^{inf}\mid+\mid T_i^{obs}-T_i^{inf}\mid\}$$
$$\times P_{m,s}\{M(S_l^{obs},S_j^{obs})\mid \Delta=\mid T_l^{obs}-T_i^{inf}\mid+\mid T_j^{obs}-T_i^{inf}\mid\}$$
$$\times P_{m,s}\{M(S_l^{obs},S_i^{obs})\mid \Delta=\mid T_l^{obs}-T_l^{inf}\mid+\mid T_i^{obs}-T_l^{inf}\mid\}$$
$$\times P_{m,s}\{M(S_l^{obs},S_k^{obs})\mid \Delta=\mid T_l^{obs}-T_i^{inf}\mid+\mid T_k^{obs}-T_i^{inf}\mid\}.\quad (3)$$

Substitutes for the conditional distribution of observed genetic sequences

We tested two expressions of the conditional distribution of observed genetic sequences

$$p_{m,s}(\mathbf{S}^{obs}\mid \mathbf{T}^{obs},J,\mathbf{T}^{inf}) = p_{m,s}(S_1^{obs}\mid \mathbf{T}^{obs},J,\mathbf{T}^{inf})\prod_{i=2}^{I} p_{m,s}(S_i^{obs}\mid S_{1:(i-1)}^{obs},\mathbf{T}^{obs},J,\mathbf{T}^{inf}).$$

The expression which led to the best reconstruction of the transmission tree is given in Eq. (5) in the main text. We tested another substitute, consisting in replacing the conditional probability $p_{m,s}(S_i^{obs}\mid S_{1:(i-1)}^{obs},\mathbf{T}^{obs},J,\mathbf{T}^{inf})$ of S_i^{obs} given past sequences S_j^{obs} $(j=1,\dots,i-1)$ by the conditional probability of S_i^{obs} given the sequence $S_{J(i)}^{obs}$ of the source of i:

$$\tilde{p}_{m,s}(\mathbf{S}^{obs} \mid \mathbf{T}^{obs}, J, \mathbf{T}^{inf}) = \prod_{i=2}^{I} P_{m,s}\{M(S_i^{obs}, S_{J(i)}^{obs}) \mid \Delta = \mid T_i^{obs} - T_{J(i)}^{inf} \mid + \mid T_j^{obs} - T_{J(i)}^{inf} \mid\}.$$

Distributions of locations X and culling times $\mathbf{T}^{end}$ for simulations

For the 20-premise simulations, locations of premises centroids were independently and uniformly drawn in rectangular domains with sizes 22×11 km, with the first infected farm at position (0,0). Distances were comparable to those in the real datasets. For the 100-premise simulation, locations of premises centroids were independently and uniformly drawn in a five times larger rectangular domain with sizes 44×27.5 km, with the first infected farm at position (0,0). Intervals between virus detection and culling time were constant and fixed to one day for all premises in all simulations.

MCMC algorithm

We built a Monte Carlo Markov Chain to assess the posterior distribution $p(J, \mathbf{T}^{inf}, \mathbf{L}, \mathbf{D}, \theta \mid data)$. With the simplifications made in section "Model Construction", the posterior distribution can be written:

$$\begin{aligned} p(J, \mathbf{T}^{inf}, \mathbf{L}, \mathbf{D}, \theta \mid data) \propto\ & p(\mathbf{S}^{obs} \mid \mathbf{T}^{obs}, J, \mathbf{T}^{inf}) \mathbf{1}(\mathbf{T}^{obs} = \mathbf{T}^{inf} + \mathbf{L} + \mathbf{D}) \\ & \times p(J, \mathbf{T}^{inf} \mid \mathbf{L}, \alpha, \mathbf{T}^{end}, \mathbf{X}) p(\mathbf{L} \mid \beta) p(\mathbf{D} \mid \mathbf{D}^{obs}) p(\alpha, \beta). \end{aligned} \quad (4)$$

In the following, premise indices are reordered at each MCMC iteration such that they are sorted with respect to increasing infection times T_i^{inf}.

MCMC tuning

Starting values. Starting values of transmissions, times and durations $(J, \mathbf{T}^{inf}, \mathbf{L}, \mathbf{D})$ were chosen to satisfy the following timing constraints:

$$\begin{aligned} & 0 \leq T_1^{inf} \leq \min\{\mathbf{T}^{end}\} \\ & T_i^{inf} \leq T_i^{inf} + L_i \leq T_i^{inf} + L_i + D_i = T_i^{obs} \leq T_i^{end} \qquad \forall i = 1, \ldots, I \\ & T_{J(i)}^{inf} + L_{J(i)} \leq T_i^{inf} \leq T_{J(i)}^{end}, \qquad \forall i = 2, \ldots, I. \end{aligned} \quad (5)$$

When possible, D_i was fixed at D_i^{obs}. Arbitrary starting values leading to a finite value of the posterior probability $p(J, \mathbf{T}^{inf}, \mathbf{L}, \mathbf{D}, \theta \mid data)$ were used for parameters α and β.

Values of fixed parameters for the cases considered. The sequence length and substitution rate were $s = 8176$ and $m = 2.076 \times 10^{-5}$for the Darlington and 2007 datasets, and $s = 8000$ and $m = 10^{-4}$ in the simulated dataset. The lower bound of infection times was fixed at $t_0 = -5$ in all the cases. Vague prior distributions were used for α and β: we fixed $a = (100, 100)$ and $b = (100, 100)$. The parameter related to the uncertainty of D_i^{obs}, d, was set to 0.5.

Proposal distributions. In the following, the star * is used to denote proposed values.

In order to update $J(i)$, the proposal distribution $q_i(J^* \mid J)$ is different in the case of the first infected premise $i = 1$, from the other premises $i > 1$:

- the first infected premise $i = 1$ is permuted with the second infected premise $i = 2$ (if several premises are infected at time T_2^{inf}, one of these premises is randomly and uniformly selected). In order to maintain the consistency of the transmission tree, we permuted T_1 and T_2, L_1 and L_2, and modified D_1 and D_2 to satisfy the equation $\mathbf{T}^{obs} = \mathbf{T}^{inf} + \mathbf{L} + \mathbf{D}$: $J^*(1) = J(2)$, $J^*(2) = J(1) = 0$, $T_1^{inf*} = T_2^{inf}$, $T_2^{inf*} = T_1^{inf}$, $L_1^* = L_2$, $L_2^* = L_1$, $D_1^* = T_1^{obs} - (T_1^{inf*} + L_1^*)$ and $D_2^* = T_2^{obs} - (T_2^{inf*} + L_2^*)$.
- for $i > 1$, a candidate value $J^*(i)$ for $J(i)$ was drawn uniformly among possible source premises satisfying constraints (5). All premises infected by i remain infected by i.

The proposal distribution $q_i(\mathbf{T}^{inf*} \mid \mathbf{T}^{inf})$ for infection time T_i^{inf} was chosen as a truncated normal distribution:

$$T_i^{inf*} \sim \text{Truncated Normal}(T_i^{inf}, \sigma_T^2, T_i^{min}, T_i^{max}),$$

where $\sigma_T^2 = 100$, $T_i^{min} = t_0 = -5$ and $T_i^{max} = \min\{\{T_k^{inf} : J(k) = i\}, T_i^{obs} - D_i\}$ if $i = 1$, $T_i^{min} = T_{J(i)}^{inf} + L_{J(i)}$ and $T_i^{max} = \min\{\{T_k^{inf} : J(k) = i\}, T_i^{obs} - D_i, T_{J(i)}^{end}\}$ if $i > 1$.

In order to maintain the consistency of the transmission tree, latency duration L_i was modified to satisfy the equation $\mathbf{T}^{obs} = \mathbf{T}^{inf} + \mathbf{L} + \mathbf{D}$: $L_i^* = T_i^{obs} - T_i^{inf*} - D_i$.

The proposal distribution $q_i(\mathbf{D}^* \mid \mathbf{D})$ for duration from infectiousness to detection D_i was chosen as a truncated normal distribution:

$$D_i^* \sim \text{Truncated Normal}(D_i, \sigma_D^2, D_i^{min}, D_i^{max}),$$

where $\sigma_D^2 = 1$, $D_i^{min} = \max\{0, \{T_i^{obs} - T_k^{inf} : J(k) = i\}\}$ and $D_i^{max} = T_i^{obs} - T_i^{inf}$. In order to maintain the consistency of the transmission tree, latency durations L_i were modified to satisfy the equation $\mathbf{T}^{obs} = \mathbf{T}^{inf} + \mathbf{L} + \mathbf{D}$: $L_i^* = T_i^{obs} - T_i^{inf} - D_i^*$.

The proposal distributions $q(\alpha^* \mid \alpha)$ and $q(\beta^* \mid \beta)$ for parameter vectors $\alpha = (\alpha_1, \alpha_2)$ and $\beta = (\beta_1, \beta_2)$ were chosen as bivariate log-normal distributions:

$$\alpha^* \sim \text{Log-Normal}(\log \alpha, \Sigma_\alpha)$$
$$\beta^* \sim \text{Log-Normal}(\log \beta, \Sigma_\beta),$$

with $\Sigma_\alpha = \left(\begin{smallmatrix} 1 & 0 \\ 0 & 1 \end{smallmatrix}\right)$ and $\Sigma_\beta = \left(\begin{smallmatrix} 1 & 0 \\ 0 & 1 \end{smallmatrix}\right)$.

Acceptance probabilities

At each iteration of the algorithm, variables were sequentially updated with the following acceptance probabilities.

The proposal distribution for $J(1)$ is symmetric. Thus, the proposed vector of values $(J^*(1), J^*(2), T_1^{inf*}, T_2^{inf*}, L_1^*, L_2^*, D_1^*, D_2^*)$ is accepted with probability:

$$\min\left\{0, \frac{p(\mathbf{S}^{obs} \mid \mathbf{T}^{obs}, J^*, \mathbf{T}^{inf*})p(J^*, \mathbf{T}^{inf*} \mid \mathbf{L}^*, \alpha, \mathbf{T}^{end}, \mathbf{X})p(\mathbf{L}^* \mid \beta)p(\mathbf{D}^* \mid \mathbf{D}^{obs})}{p(\mathbf{S}^{obs} \mid \mathbf{T}^{obs}, J, \mathbf{T}^{inf})p(J, \mathbf{T}^{inf} \mid \mathbf{L}, \alpha, \mathbf{T}^{end}, \mathbf{X})p(\mathbf{L} \mid \beta)p(\mathbf{D} \mid \mathbf{D}^{obs})}\right\},$$

where $(J^*, \mathbf{T}^{inf*}, \mathbf{L}^*, \mathbf{D}^*)$ is equal to $(J, \mathbf{T}^{inf}, \mathbf{L}, \mathbf{D})$ except that $(J(1), J(2), T_1^{inf}, T_2^{inf}, L_1, L_2, D_1, D_2)$ is replaced by $(J^*(1), J^*(2), T_1^{inf*}, T_2^{inf*}, L_1^*, L_2^*, D_1^*, D_2^*)$.

The proposal distribution for $J(i)$, $i > 1$, is symmetric. Thus, the proposed value $J^*(i)$ is accepted with probability:

$$\min\left\{0, \frac{p(\mathbf{S}^{obs} \mid \mathbf{T}^{obs}, J^*, \mathbf{T}^{inf})p(J^*, \mathbf{T}^{inf} \mid \mathbf{L}, \alpha, \mathbf{T}^{end}, \mathbf{X})}{p(\mathbf{S}^{obs} \mid \mathbf{T}^{obs}, J, \mathbf{T}^{inf})p(J, \mathbf{T}^{inf} \mid \mathbf{L}, \alpha, \mathbf{T}^{end}, \mathbf{X})}\right\},$$

where J^* is equal to J except that $J(i)$ is replaced by $J^*(i)$.

The proposed vector of values (T_i^{inf*}, L_i^*) is accepted with probability:

$$\min\left\{0, \frac{p(\mathbf{S}^{obs} \mid \mathbf{T}^{obs}, J, \mathbf{T}^{inf*})p(J, \mathbf{T}^{inf*} \mid \mathbf{L}^*, \alpha, \mathbf{T}^{end}, \mathbf{X})p(\mathbf{L}^* \mid \beta)q_i(\mathbf{T}^{inf} \mid \mathbf{T}^{inf*})}{p(\mathbf{S}^{obs} \mid \mathbf{T}^{obs}, J, \mathbf{T}^{inf})p(J, \mathbf{T}^{inf} \mid \mathbf{L}, \alpha, \mathbf{T}^{end}, \mathbf{X})p(\mathbf{L} \mid \beta)q_i(\mathbf{T}^{inf*} \mid \mathbf{T}^{inf})}\right\},$$

where $(\mathbf{T}^{inf*}, \mathbf{L}^*)$ is equal to $(\mathbf{T}^{inf}, \mathbf{L})$ except that (T_i^{inf}, L_i) is replaced by (T_i^{inf*}, L_i^*).

The proposed vector of values (D_i^*, L_i^*) is accepted with probability:

$$\min\left\{0, \frac{p(J, \mathbf{T}^{inf} \mid \mathbf{L}^*, \alpha, \mathbf{T}^{end}, \mathbf{X})p(\mathbf{L}^* \mid \beta)p(\mathbf{D}^* \mid \mathbf{D}^{obs})q_i(\mathbf{D} \mid \mathbf{D}^*)}{p(J, \mathbf{T}^{inf} \mid \mathbf{L}, \alpha, \mathbf{T}^{end}, \mathbf{X})p(\mathbf{L} \mid \beta)p(\mathbf{D} \mid \mathbf{D}^{obs})q_i(\mathbf{D}^* \mid \mathbf{D})}\right\},$$

where $(\mathbf{D}^*, \mathbf{L}^*)$ is equal to $(\mathbf{D}, \mathbf{L})$ except that (D_i, L_i) is replaced by (D_i^*, L_i^*).

The proposed vector of values α^* is accepted with probability:

$$\min\left\{0, \frac{p(J, \mathbf{T}^{inf} \mid \mathbf{L}, \alpha^*, \mathbf{T}^{end}, \mathbf{X})p(\alpha^*, \beta)q(\alpha \mid \alpha^*)}{p(J, \mathbf{T}^{inf} \mid \mathbf{L}, \alpha, \mathbf{T}^{end}, \mathbf{X})p(\alpha, \beta)q(\alpha^* \mid \alpha)}\right\}.$$

The proposed vector of values β^* is accepted with probability:

$$\min\left\{0, \frac{p(\mathbf{L} \mid \beta^*)p(\alpha, \beta^*)q(\beta \mid \beta^*)}{p(\mathbf{L} \mid \beta)p(\alpha, \beta)q(\beta^* \mid \beta)}\right\}.$$

Performance of the estimation algorithm

Using the series of simulations for 20 premises described in the main text, we assessed the ability of our method to estimate unobserved time variables and parameters, namely infection times (T_i^{inf}), infectiousness times ($T_i^{inf} + L_i$), the source strength (α_1), the dispersion parameter ($2\alpha_2$), the latency mean (β_1) and the latency standard deviation (β_2). We considered the coverages of the true values by the 95%-credibility intervals, listed in Table S1. The coverage of infection and infectiousness times is high, ranging from 0.78 to 0.95, while the coverage of parameters is more heterogeneous and depends on the characteristics of the epidemics (e.g. number of farms and parameter values). In particular, potentially identifiability issues could have affected the lower coverage values.

Darlington cluster: comparison with the results of Cottam et al. 2008

The analysis of Ref. [3] indicated that two of these 15 premises (A, N) were infected from a second source outwith our sample. In order to maintain the assumption of a single introduction required by our model, we initally applied our inference scheme on the 13 remaining premises. We inferred that premise B acted as a "hub" of the outbreak, infecting 7 premises (see Fig. S8), in contrast with Cottam *et al.* [3], where the role of the hub was assigned to premise K, which was inferred as a source for B. The sequences collected on the premises infected by the hub are indeed closer to K than to B thus genetic data support K as the hub. However, the lesion age estimates combined with the observation times indicate that K and B became infectious on the same day and, consequently, both K→B and B→K transmissions were unlikely. Thus, the timing data support the hypothesis that premises B and K were infected independently approximately at the same time. To compare our results with those of Ref. [3] on a cluster infected by a single introduction, we removed B from the dataset and re-estimated the quantities of interest, thus applying our method to 12 premises in total.

Our estimation, detailed in the main text (result section on the 2001 FMDV epidemic) found only two chains of transmissions of length greater than two (K→O→(M,D) and K→F→G→I→J), whereas ref. [3] found more long chains: K→O→M→D, K→O→P, K→O→C and K→L→E. Regarding the first one, the observed timing and the estimated lesion ages suggest again that M and D became infectious almost simultaneously and, therefore, M→D or D→M transmissions are unlikely. On what concerns the K→O→P and K→O→C cases, we note that P and C are closer genetically to K than to O, supporting the possibility of a more direct link between K and P, and K and C, respectively. Finally, the large number of nucleotide substitutions between L and E in a relatively short time makes the K→L→E chain very unlikely, leading our method to rather support the double transmission K→(L,E). Further information about the inferred trees and posterior distributions of other model parameters is provided in Figs. S10–S11.

Application to a simulation with 100 premises

In order to test our inference on a larger dataset, we used our model to simulate an outbreak infecting 100 premises. The locations of these premises are randomly distributed in a 44×27.5km, so that their spatial density is the same as in the test dataset used in the main text. The model was fitted to the observable data: for each premise i, the time T_i^{obs} at which the virus was detected, an 8000bp DNA sequence S_i^{obs} sampled at T_i^{obs}, an assessment of the lesion age D_i^{obs}, and the time T_i^{end} at which the premise was culled (see Fig. 1 in the main text for a visualisation).

In Fig. S15, the size of the dots corresponds to the posterior probabilities of pairwise transmissions, while the circles represent the true transmissions as they occurred in the simulation. Fig. S16 shows the transmissions with highest posterior probability (solid lines)

together with the "true" transmissions (dashed lines). 89 of the 99 transmissions were accurately reconstructed; most of the incorrectly renconstructed transmissions happened either at the very beginning of the outbreak or in clusters of farms very closely located. Both situations are particularly ambiguous, with several scenarios having very similar likelihoods, which can be distinguished only in presence of extremely precise data. We notice however that the directions of the uncorrectly inferred transmissions is compatible with the overall spreading of the epidemics (started in the lower-left corner). Given the extremely fast pace of this simulated outbreak and the high density of the premises, this situation should be considered as a worst-case scenario of the real case.

Finally, we notice that the posterior probabilities for the mean latency duration and the mean transmission distance (Fig. S17) have a similar shape to those obtained for the 20 premise simulation (Fig. 2), but their width is much smaller. In the case of the mean latency duration, the true value of this parameter is not contained in the 95% confidence interval of the corresponding posterior distribution. This is probably due to the "extreme" character of the epidemics, as described above. However, given the small width of this posterior distribution, the difference between the true value of the parameter and the median of the posterior is less than a day, which in absolute terms is less than what was obtained for the 20 premise simulation.

References

[1] M. Clement, D. Posada and K.A. Crandall, *TCS: a computer program to estimate gene genealogies*, Molecular Ecology, 9 (2000), pp. 1657–1659.

[2] E.M. Cottam, J. Wadsworth, A.E. Shaw, R.J. Rowlands and N.J. Knowles, *Transmission Pathways of Foot-and-Mouth Disease Virus in the United Kingdom in 2007*, PLoS Pathog., 4 (2008), e1000050.

[3] E.M. Cottam, G. Thébaud, J. Wadsworth, J. Gloster, L. Mansley, D.J. Paton, D.P. King, and D.T. Haydon, *Integrating genetic and epidemiological data to determine transmission pathways of foot-and-mouth disease virus*, Proc. R. Soc. B, 275 (2008), pp. 887–895.
